# Effects of Physical Activity Interventions on Anthropometric, Body Composition, and Physical Fitness Indicators in Chilean Children and Adolescents: Systematic Review and Meta-Analysis

**DOI:** 10.3390/children13101368

**Published:** 2026-10-08

**Authors:** Paula Lavalle-Mellado, Andrés Godoy-Cumillaf, Josivaldo de Souza-Lima, Maribel Parra-Saldias, Daniel Duclos-Bastias, Claudio Farias-Valenzuela, Eugenio Merellano-Navarro, Valentina Díaz-Goñi, Bruno Bizzozero-Peroni

**Affiliations:** 1 Grupo de Investigación en Educación Física, Salud y Calidad de Vida (EFISAL), Facultad de Educación, Universidad Autónoma de Chile, Temuco 4780000, Chile; paula.lavalle@cloud.uautonoma.cl; 2 Facultad de Educación y Ciencias Sociales, Instituto del Deporte y Bienestar, Universidad Andres Bello, Las Condes, Santiago 7550000, Chile; josivaldo.desouza@unab.cl; 3 Departamento de Educación Física, Deporte y Recreación, Universidad de Atacama, Copiapó 1530000, Chile; maribel.parra@uda.cl; 4 iGEO, Escuela de Educación Física, Facultad de Filosofía y Educación, Pontificia Universidad Católica de Valparaíso, Valparaíso 2340025, Chile; 5 METIS Research Lab, Facultad de Negocios y Tecnología, Universidad Alfonso X el Sabio (UAX), 28691 Madrid, Spain; 6 Escuela de Ciencias de la Actividad Física, Universidad de Las Américas, Santiago 8320000, Chile; claudio.farias.valenzuela@edu.udla.cl; 7 Department of Physical Activity Sciences, Faculty of Education Sciences, Universidad Católica del Maule, Talca 3530000, Chile; emerellano@ucm.cl; 8 Health and Social Research Center, Universidad de Castilla-La Mancha, 16002 Cuenca, Spain; valentina.diaz@uclm.es (V.D.-G.); bruno.bizzozero@uclm.es (B.B.-P.); 9 Faculty of Education, Universidad de Castilla-La Mancha, 14071 Ciudad Real, Spain; 10 Higher Institute of Physical Education, Universidad de la República, Maldonado 20000, Uruguay

**Keywords:** adiposity, cardiorespiratory fitness, childhood obesity, cardiometabolic health, muscular fitness, school-based intervention

## Abstract

**Highlights:**

**What are the main findings?**

- Physical activity interventions were associated with improvements in cardiorespiratory fitness among Chilean children and adolescents.
- Reductions were observed in body fat percentage and triceps skinfold thickness, whereas no clear changes were found in BMI and waist circumference.

**What are the implications of the main findings?**

- The very low certainty of the evidence warrants cautious interpretation of the observed improvements in cardiorespiratory fitness and adiposity outcomes.
- Future school-based programs should assess cardiorespiratory fitness and specific measures of adiposity and body composition alongside conventional anthropometric indicators.

**Abstract:**

**Background/Objectives**: Physical activity interventions are widely implemented to promote health during childhood and adolescence; however, their effects on anthropometric, body composition, and physical fitness outcomes among Chilean pediatric populations remain unclear. This systematic review and meta-analysis aimed to determine the effects of physical activity interventions on these outcomes in Chilean children and adolescents. **Methods**: PubMed, Scopus, and Web of Science were systematically searched from inception to 30 May 2026, supplemented by Google Scholar and reference-list screening. The review followed the PRISMA 2020 guidelines and Cochrane recommendations and was registered in PROSPERO (CRD42023398677). Random-effects meta-analyses were conducted using standardized mean differences (SMDs) and 95% confidence intervals (CIs). **Results**: Eighteen studies involving participants aged 2–19 years were included, of which 16 contributed data to the meta-analysis. Pooled estimates favored physical activity interventions for cardiorespiratory fitness (SMD = 0.76; 95% CI: 0.48 to 1.04; I^2^ = 73.2%), body fat percentage (SMD = −0.83; 95% CI: −1.37 to −0.29; I^2^ = 84.9%), and triceps skinfold thickness (SMD = −0.14; 95% CI: −0.20 to −0.08; I^2^ = 0%). No clear difference was observed for waist circumference (SMD = −0.05; 95% CI: −0.14 to 0.05) or for BMI in studies with a concurrent control group (SMD = −0.37; 95% CI: −0.79 to 0.05) and in uncontrolled before–after studies (SMD = −0.13; 95% CI: −0.34 to 0.09). Between-study heterogeneity was substantial to considerable for most outcomes, and the certainty of evidence was very low for all meta-analyzed outcomes. **Conclusions**: Physical activity interventions were associated with more favorable pooled estimates for cardiorespiratory fitness and specific measures of adiposity among Chilean children and adolescents, whereas no clear differences were observed in BMI or waist circumference. However, substantial between-study heterogeneity and very low certainty of evidence limit confidence in these findings and warrant cautious interpretation. Further well-designed randomized controlled trials are needed to clarify these associations.

## 1. Introduction

Childhood excess weight represents one of the major global public health challenges due to its high prevalence and the metabolic, cardiovascular, and psychosocial consequences that emerge early in life [1]. The World Health Organization estimates that the prevalence of childhood overweight and obesity continues to increase in most countries, particularly in middle- and high-income regions [2]. In Latin America, and particularly in Chile, the prevalence of excess weight among children is among the highest in the region, representing a major challenge for public policies aimed at promoting healthy lifestyles [3]. Recent national surveillance data further illustrates the magnitude of this problem. According to the 2024 JUNAEB Nutritional Map, 50.9% of the students assessed had excess weight, including 27.0% with overweight, 17.9% with obesity, and 6.0% with severe obesity [4]. Consequently, identifying effective strategies to prevent and manage excess weight during childhood is a priority for both health and education systems. Excess adiposity during childhood and adolescence is associated with metabolic and cardiovascular abnormalities, hypertension, type 2 diabetes mellitus, certain types of cancer, and a greater likelihood of obesity persisting into adulthood, thereby increasing the risk of premature morbidity and mortality [5,6,7].

The assessment of nutritional status in children and adolescents has traditionally relied on anthropometric indicators such as body mass index (BMI), owing to its ease of measurement and usefulness for classifying weight status in epidemiological studies [8,9]. However, BMI has limitations in accurately reflecting changes in body composition during growth, as it does not distinguish between fat mass and fat-free mass [10]. In this context, indicators such as waist circumference and triceps skinfold thickness complement nutritional assessment by providing more specific estimates of central and subcutaneous adiposity, which are closely associated with cardiometabolic risk in pediatric populations [11,12]. Beyond body composition, physical fitness is an important marker of health during childhood and adolescence. In particular, cardiorespiratory fitness has been recognized as one of the most robust predictors of current and future cardiovascular and metabolic health, showing inverse associations with obesity, metabolic syndrome, type 2 diabetes, and premature mortality [13].

Several studies have shown that children and adolescents with higher levels of cardiorespiratory fitness exhibit a more favorable cardiometabolic profile, regardless of their nutritional status [14,15,16]. In Chilean pediatric populations, an inverse association between cardiorespiratory fitness and several indicators of adiposity has also been reported, further supporting its usefulness as a comprehensive marker of health during childhood [17].

Physical activity is one of the primary non-pharmacological strategies for preventing and managing excess weight during childhood and adolescence [18,19]. However, physical activity levels remain low among Chilean children and adolescents. The 2022 Chilean Report Card showed that only 37% of children and adolescents aged 5–17 years reported engaging in at least 60 min of physical activity on four or more days per week, while only 24% of adolescents aged 13–17 years met the recommendation of no more than 2 h of recreational screen time per day [20]. Available evidence indicates that physical activity interventions may improve body composition, physical fitness, cardiometabolic health, and various indicators of physical and psychological well-being [21]. However, findings regarding anthropometric outcomes, particularly BMI, remain inconsistent across studies. This variability may be partly explained by the limited ability of BMI to distinguish between fat mass and fat-free mass, as well as differences in intervention intensity, duration, and design, and in the characteristics of the populations studied. Other outcomes, including waist circumference, skinfold thickness, and cardiorespiratory fitness, may respond differently depending on the intensity, duration, and characteristics of the interventions.

Although numerous interventions aimed at increasing physical activity and improving health outcomes among children and adolescents have been implemented in Chile [15,22,23,24], the available evidence remains fragmented and has not been specifically synthesized within the national context. A country-specific synthesis is relevant because the design and implementation of these interventions are closely linked to the Chilean educational and public health context, with most programs delivered in school settings and some embedded within national or local health-promotion initiatives. Previous evidence syntheses have examined studies from different Latin American countries [25,26]. These countries vary in their educational systems, public health policies, intervention delivery, and population characteristics, while the syntheses have also included interventions combining physical activity with nutritional components. Consequently, regional estimates may not fully reflect the characteristics or effects of physical activity interventions implemented in Chile. In addition, previous reviews have frequently focused on BMI or obesity-related outcomes. However, anthropometric indicators should be interpreted alongside more specific measures of adiposity and physical fitness to provide a more comprehensive assessment of intervention effects. Therefore, a Chile-specific synthesis can provide a more contextually relevant assessment of the available evidence and identify outcomes that may be particularly responsive to physical activity interventions in this population. Accordingly, this systematic review and meta-analysis aimed to determine the effects of physical activity interventions implemented in Chilean children and adolescents on anthropometric, body composition, and physical fitness outcomes.

## 2. Materials and Methods

### 2.1. Study Design

This systematic review and meta-analysis were conducted in accordance with the Preferred Reporting Items for Systematic Reviews and Meta-Analyses (PRISMA) 2020 statement [27] and the methodological guidance provided in the Cochrane Handbook for Systematic Reviews of Interventions [28]. The completed PRISMA 2020 checklist is provided in the Supplementary Materials (Table S1). The study protocol was prospectively registered in the International Prospective Register of Systematic Reviews (PROSPERO; registration number CRD42023398677) and published in a peer-reviewed journal to enhance methodological transparency and prespecify the review methods [29]. Some amendments to the published protocol were made during the review, with their rationale detailed in Table S1.

The literature search, study selection, data extraction, and risk-of-bias assessment were independently conducted by two previously trained reviewers (PL-M and AG-C). Any discrepancies arising at any stage of the review process were resolved through discussion and consensus. When consensus could not be reached, a third reviewer (BB-P) was consulted to make the final decision.

Prior to conducting the review, the eligibility criteria, search strategy, outcomes of interest, data extraction procedures, risk of bias assessment, and statistical analysis plan were explicitly predefined.

### 2.2. Search Strategy

A systematic literature search was conducted in the electronic databases PubMed, Web of Science, and Scopus, from database inception to 30 May 2026. The search combined terms related to physical activity, children and adolescents, Chile, and anthropometric and physical fitness outcomes. Database-specific fields were used, and no language restrictions were applied. The complete database-specific search strategies, including field tags and search syntax, are provided in Table S2.

To maximize search sensitivity and reduce the risk of missing potentially eligible studies, supplementary searches were also conducted in Google Scholar, where the first 200 records ranked by relevance were screened. In addition, the reference lists of all included studies were manually screened to identify further potentially eligible studies. Details of the Google Scholar search procedure and supplementary search methods are also provided in Table S2.

### 2.3. Eligibility Criteria

Studies were selected according to the PICOS framework, which was used to explicitly define the prespecified inclusion and exclusion criteria.

Participants: Studies involving children and adolescents aged 2–19 years residing in Chile were eligible, regardless of sex, educational level, or nutritional status.

Intervention: Eligible interventions were those designed to increase physical activity levels through school-based programs, increased time allocated to Physical Education classes, structured exercise programs, high-intensity interval training (HIIT), sports activities, active recreational programs, or multicomponent interventions in which physical activity was a core, explicitly planned component. For multicomponent interventions, physical activity was considered a core component when participants received a structured or systematically promoted physical activity exposure, such as supervised exercise sessions, additional or modified Physical Education classes, or a defined school-based physical activity program. Interventions in which physical activity was limited to general advice or constituted only an incidental component were not eligible.

Comparison: Comparison conditions included groups that continued with the usual school curriculum, traditional Physical Education classes, or no additional intervention. For uncontrolled before–after studies, measurements obtained before and after the intervention were considered for comparison.

Outcomes: Studies were eligible if they reported at least one of the following outcomes: anthropometric (i.e., body mass index [BMI], waist circumference, and triceps skinfold thickness), body composition (i.e., body fat and lean mass), and/or health-related physical fitness (i.e., cardiorespiratory fitness, muscular strength, speed-agility, and flexibility) indicators.

Randomized controlled trials, non-randomized controlled studies, and uncontrolled before–after studies were eligible for inclusion.

Qualitative studies, systematic reviews, study protocols, conference abstracts, and editorials were excluded. Studies that did not evaluate physical activity-based interventions or did not report specific data for at least one of the outcomes considered in this systematic review were also excluded.

### 2.4. Study Selection

After duplicate records were removed, two reviewers independently conducted the study selection process by screening titles, abstracts, and full-text articles. Potentially eligible studies were assessed in full to determine whether they met the predefined inclusion criteria. Any disagreements between the reviewers were resolved through consensus and, when necessary, by consultation with a third reviewer. The study identification, screening, eligibility, and inclusion process was documented using the PRISMA flow diagram.

### 2.5. Data Extraction

Data were extracted using a standardized data extraction form specifically developed for this review. For each included study, information was collected on the first author, year of publication, country, study design, sample size, participant characteristics (age and sex), intervention characteristics (duration, frequency, intensity, and components), anthropometric, body composition, and health-related physical fitness outcomes, as well as the statistical data required for the quantitative synthesis. For each intervention, we additionally extracted whether the intervention was physical-activity-only or multicomponent, the dose of the physical activity component, and the type and dose of any non-physical-activity components (e.g., nutrition education, psychological support, or health education). For multicomponent interventions, the proportion of scheduled intervention time attributable to physical activity was calculated when sufficient component-specific duration data were available. When one or more components were not reported in comparable time units or involved non-time-based strategies, this proportion was classified as not calculable from the published report.

Specifically, sample sizes, means, and standard deviations for pre- and post-intervention measurements were extracted for both the intervention and control groups. When available, mean changes from pre- to post-intervention and their corresponding standard deviations were also extracted directly.

### 2.6. Risk of Bias Assessment

Risk of bias was assessed according to study design and for the outcomes contributing to the quantitative synthesis. The cluster-randomized controlled trial was evaluated using the Revised Cochrane Risk of Bias Tool (RoB 2) for cluster-randomized trials (RoB 2) [30], which assesses six domains: bias arising from the randomization process, bias arising from the timing of identification and recruitment of participants, bias due to deviations from intended interventions, bias due to missing outcome data, bias in the measurement of outcomes, and bias in the selection of the reported results. Each domain was judged as low risk of bias, some concerns, or high risk of bias, and an overall risk-of-bias judgment was subsequently assigned to each study in accordance with the methodological guidance of the RoB 2 tool [30].

Non-randomized controlled trials and uncontrolled before–after studies were assessed using the Risk Of Bias In Non-randomized Studies of Interventions (ROBINS-I) tool [31], which evaluates seven domains: bias due to confounding, bias in the selection of participants, bias in the classification of interventions, bias due to deviations from intended interventions, bias due to missing data, bias in the measurement of outcomes, and bias in the selection of the reported results. For uncontrolled before–after studies, the confounding domain explicitly considered secular trends, maturation, regression to the mean, and other time-varying factors that could explain pre–post changes independently of the intervention. The overall risk of bias was classified as low, moderate, serious, or critical, in accordance with the ROBINS-I recommendations [31].

### 2.7. Data Synthesis and Statistical Analysis

#### 2.7.1. Effect Measure

The effects of the interventions on anthropometric, body composition, and physical fitness outcomes were estimated using the standardized mean difference (SMD) with corresponding 95% confidence intervals (CIs). The magnitude of the effect sizes was interpreted according to Cohen’s criteria as small (SMD = 0.20), moderate (SMD = 0.50), or large (SMD = 0.80) [32]. The use of SMDs enabled the pooling of studies that assessed the same outcome using different protocols and measurement instruments.

For studies with a concurrent control group, effect estimates represented the between-group difference in change from baseline (Δ intervention − Δ control, where Δ = post-intervention—pre-intervention). For uncontrolled before–after studies, standardized mean changes were calculated from paired pre- and post-intervention measurements and synthesized separately from studies with a concurrent control group. Sampling variances accounted for the within-participant correlation between pre- and post-intervention measurements. Because pre–post correlations were not reported or could not be derived from the original studies, a correlation of r = 0.50 was assumed in the primary analysis. Sensitivity analyses using alternative correlations of r = 0.30 and r = 0.70 were conducted to assess the robustness of the pooled estimate to this assumption, in accordance with the methodological recommendations of the Cochrane Handbook for Systematic Reviews of Interventions [28]. Outcome direction was harmonized so that negative SMD values indicated favorable reductions in anthropometric and adiposity outcomes, whereas positive SMD values indicated favorable improvements in physical fitness outcomes. Sample sizes, means, and standard deviations were extracted for the relevant comparison groups or time points. Further details on the original outcome metrics, direction of benefit, data transformations and harmonization, and definitions of the effect contrasts used for each outcome are provided in Table S3.

#### 2.7.2. Quantitative Synthesis

Given the anticipated methodological heterogeneity across studies arising from differences in population characteristics, intervention duration, physical activity modalities, and outcome assessment methods, pooled effect sizes were estimated using a random-effects model, which was considered the most appropriate approach for this body of evidence. Between-study heterogeneity was assessed using the I^2^ statistic and interpreted as not important (0–30%), moderate (30–60%), substantial (60–75%), or considerable (>75%), in accordance with the recommendations of the Cochrane Handbook [28]. Additionally, 95% prediction intervals were calculated to reflect the expected range of effects that might be observed in a future study [33].

Separate meta-analyses were conducted for outcomes reported by at least two studies. BMI was analyzed separately for studies with a concurrent control group and uncontrolled before–after studies. Waist circumference, triceps skinfold thickness, body fat percentage, and cardiorespiratory fitness were meta-analyzed using studies with a concurrent control group. Leave-one-out sensitivity analyses were performed to assess the robustness of the pooled estimates [28].

Results were presented using forest plots, and statistical significance was set at *p* < 0.05. All statistical analyses were performed using R software (version 4.4.2; R Foundation for Statistical Computing) with the meta package [34].

#### 2.7.3. Additional Methodological Considerations

When studies did not provide sufficient data for quantitative synthesis, the corresponding authors were contacted to request the missing information [22,35]. The authors of these studies did not respond, and because the required data could not be obtained, the studies were excluded from the meta-analysis but retained in the qualitative synthesis. When results were reported only graphically [36], data were extracted using the online WebPlotDigitizer software (version 4.8; Ankit Rohatgi, Pacifica, CA, USA) [37]. Finally, when studies reported results separately according to participants’ sex or the test used to assess the same outcome [23,38,39,40], these data were combined to obtain a single effect estimate per study and to avoid double-counting participants.

### 2.8. Certainty of Evidence Assessment

The certainty of evidence was assessed for each meta-analyzed outcome using the Grading of Recommendations Assessment, Development and Evaluation (GRADE) framework [41]. The assessment considered risk of bias, inconsistency, indirectness, imprecision, and other considerations. Risk-of-bias judgments were informed by the RoB 2 and ROBINS-I assessments. Inconsistency was evaluated considering the magnitude and direction of study-specific effects, the I^2^ statistic, and 95% prediction intervals. Indirectness was assessed in relation to the population, intervention, comparator, and outcomes specified in the review question. Imprecision was evaluated from the 95% confidence interval around the pooled effect and whether it included the null and materially different interpretations of the intervention effect. Certainty of evidence was categorized as high, moderate, low, or very low. Detailed judgments and explanations are provided in Table S4.

## 3. Results

### 3.1. Literature Search

**Figure 1:**
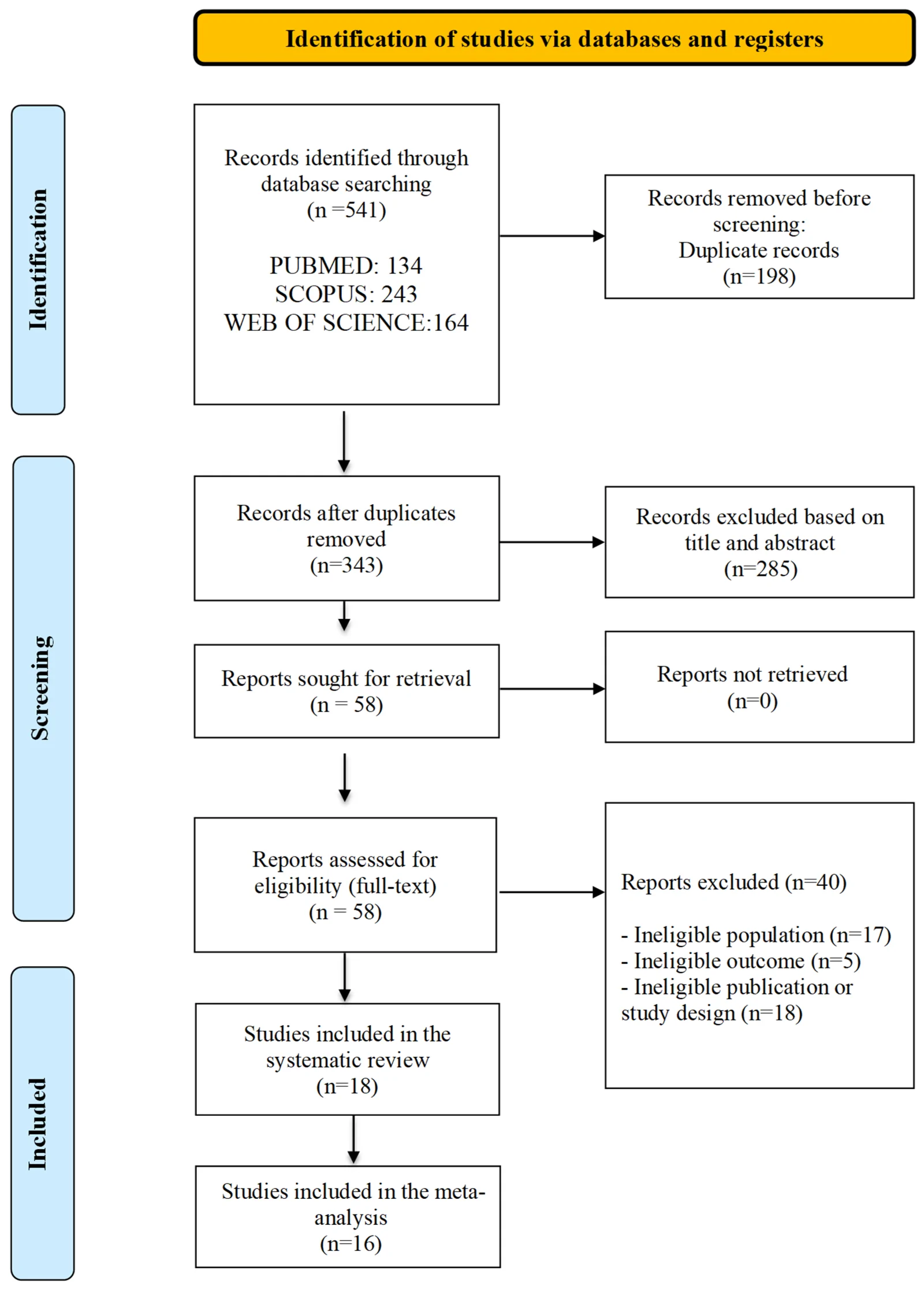
PRISMA 2020 flow diagram.

The literature search identified a total of 541 records through PubMed (n = 134), Scopus (n = 243), and Web of Science (n = 164). After removing 198 duplicate records, 343 records were screened based on their titles and abstracts, of which 285 were excluded. The remaining 58 reports were sought for retrieval, and all were assessed for full-text eligibility. Ultimately, 18 studies met the inclusion criteria and were included in the systematic review [22,23,24,35,36,38,39,40,42,43,44,45,46,47,48,49,50,51], of which 16 provided sufficient data for inclusion in the meta-analysis (Figure 1). Studies excluded following full-text assessment, together with the corresponding reasons for exclusion, are detailed in Table S5.

### 3.2. Characteristics of the Included Studies

The general characteristics of the included studies are presented in Table 1. The 18 studies were published between 2004 and 2025 and evaluated the effects of different physical activity interventions on anthropometric, body composition, and physical fitness outcomes in Chilean children and adolescents.

Although all studies met the eligibility criteria for inclusion in the systematic review, some did not provide sufficient statistical information for specific outcomes and, therefore, could not be included in the corresponding quantitative synthesis. Specifically, studies [22] and [48] reported incomplete data that precluded the calculation of the SMD and its corresponding 95% CIs. Consequently, these studies were included in the qualitative synthesis but excluded from the meta-analyses for which sufficient data were unavailable.

From a methodological perspective, the body of evidence comprised a combination of one cluster-randomized controlled trial [39], non-randomized controlled trials [22,23,35,36,38,40,42,44,45], and uncontrolled before–after studies [22,38,41,42,43,44,45,46], reflecting the diversity of methodological approaches used to evaluate physical activity promotion programs among Chilean children and adolescents.

Sample sizes varied considerably across studies, ranging from pilot studies involving fewer than 50 participants to large population-based studies including more than 13,000 participants. Most interventions were implemented in educational settings and primarily involved school-aged children, although preschool-aged children and adolescents were also represented. Across the 18 included studies, participants ranged in age from 2 to 19 years. Among studies reporting a mean age, mean values ranged from 6.1 to 15.6 years. Among studies for which age ranges were available, the lower age limits ranged from 2 to 8 years and the upper age limits from 5 to 19 years. Age was reported heterogeneously across primary studies, either as a mean and/or as an age range (Table 1). Several studies included participants spanning more than one developmental stage, precluding a reliable classification of the overall sample into mutually exclusive preschool, school-aged childhood, and adolescent groups.

### 3.3. Intervention Characteristics

The included interventions showed considerable heterogeneity in terms of duration, frequency, intensity, and components, reflecting the diversity of strategies implemented to increase physical activity levels within the Chilean school context.

Overall, the interventions comprised school-based physical activity programs, increased time allocated to Physical Education classes, structured exercise training, high-intensity interval training (HIIT), sports and recreational activities, as well as multicomponent interventions combining physical activity with nutrition education and the promotion of healthy lifestyles.

Twelve interventions were multicomponent, whereas six consisted exclusively of structured physical activity or exercise. Among the multicomponent interventions, physical activity was combined with nutrition education, psychological support, health education, or broader school- or family-based strategies. Component-specific intervention doses and the proportion of scheduled intervention time attributable to physical activity, where calculable, are presented in Table S6.

Intervention duration ranged from five weeks to three years, while intervention frequency varied from one to five sessions per week. Activities were primarily implemented during school hours and supervised by Physical Education teachers or multidisciplinary teams.

Comparison conditions mainly consisted of the usual school curriculum, traditional Physical Education classes, or no additional intervention. In uncontrolled before–after studies, comparisons were based on measurements obtained before and after the intervention.

### 3.4. Outcomes

The included studies assessed a range of outcomes related to anthropometric indicators, body composition, and physical fitness. Among the anthropometric indicators, BMI was the most frequently assessed outcome. Seven studies with a concurrent control group provided BMI data for quantitative synthesis, while an additional seven uncontrolled before–after studies contributed to a separate meta-analysis. Four studies contributed to the waist circumference meta-analysis, and triceps skinfold thickness was evaluated in two studies. These variables were assessed using standardized anthropometric procedures, including weight and height measurements for BMI calculation, direct measurement of waist circumference, and assessment of triceps skinfold thickness.

Regarding body composition, four studies assessed body fat percentage. The methods used to estimate adiposity varied across studies, reflecting differences in assessment protocols and the characteristics of the populations examined.

Cardiorespiratory fitness was the main physical fitness outcome, with seven studies contributing to quantitative synthesis. It was evaluated using field-based tests designed to estimate cardiorespiratory fitness, including progressive running and walking tests and estimates of maximal oxygen uptake (VO_2_max). The use of different assessment protocols across studies supported the standardization of effect estimates for subsequent quantitative synthesis.

In addition to these outcomes, some studies assessed other components of health-related physical fitness, including muscular strength, speed-agility, and flexibility. However, owing to the limited number of studies reporting these outcomes and the heterogeneity of the assessment instruments used, these outcomes could not be quantitatively pooled and were therefore summarized narratively.

### 3.5. Summary of Outcomes Not Meta-Analyzed

Some components of physical fitness were assessed in a limited number of studies and were therefore summarized narratively. Regarding flexibility, one study [23] assessed lower-back flexibility and reported improvements in both the intervention and control groups (Δ = +2.4 and +3.9 cm, respectively), with no greater improvement observed in the intervention group.

Regarding speed-agility and lower-limb muscular strength, one study [45] reported significant improvements in the intervention group. Standing long-jump performance increased from 79.56 ± 23.35 to 86.83 ± 21.68 cm (*p* < 0.001; d = 0.38), while performance time in the 4 × 10 m speed-agility test decreased from 17.01 ± 1.90 to 16.63 ± 1.66 s (*p* = 0.002; d = −0.30). However, significant pre–post improvements in both outcomes were also observed in the control group, with no clear evidence of a differential effect favoring the intervention.

In an uncontrolled before–after study [49], significant pre–post increases in cardiorespiratory fitness were observed using the 6-min walk test, with walking distance increasing by 14.5 to 17.4 m among participants aged 6–19 years (*p* < 0.05). Lower-limb muscular endurance, assessed using the 30-s squat test, also increased by approximately 2.5–2.6 repetitions (*p* < 0.05). Among participants aged 2–5 years, motor performance was assessed using the standing long-jump test, with significant pre–post changes also reported.

Finally, one uncontrolled before–after study [51] reported no significant pre–post change in handgrip strength (18.0 vs. 18.5 kg; *p* = 0.413), whereas standing long-jump performance decreased from 117.0 to 115.5 cm (*p* < 0.001).

### 3.6. Meta-Analysis Results

**Figure 2:**
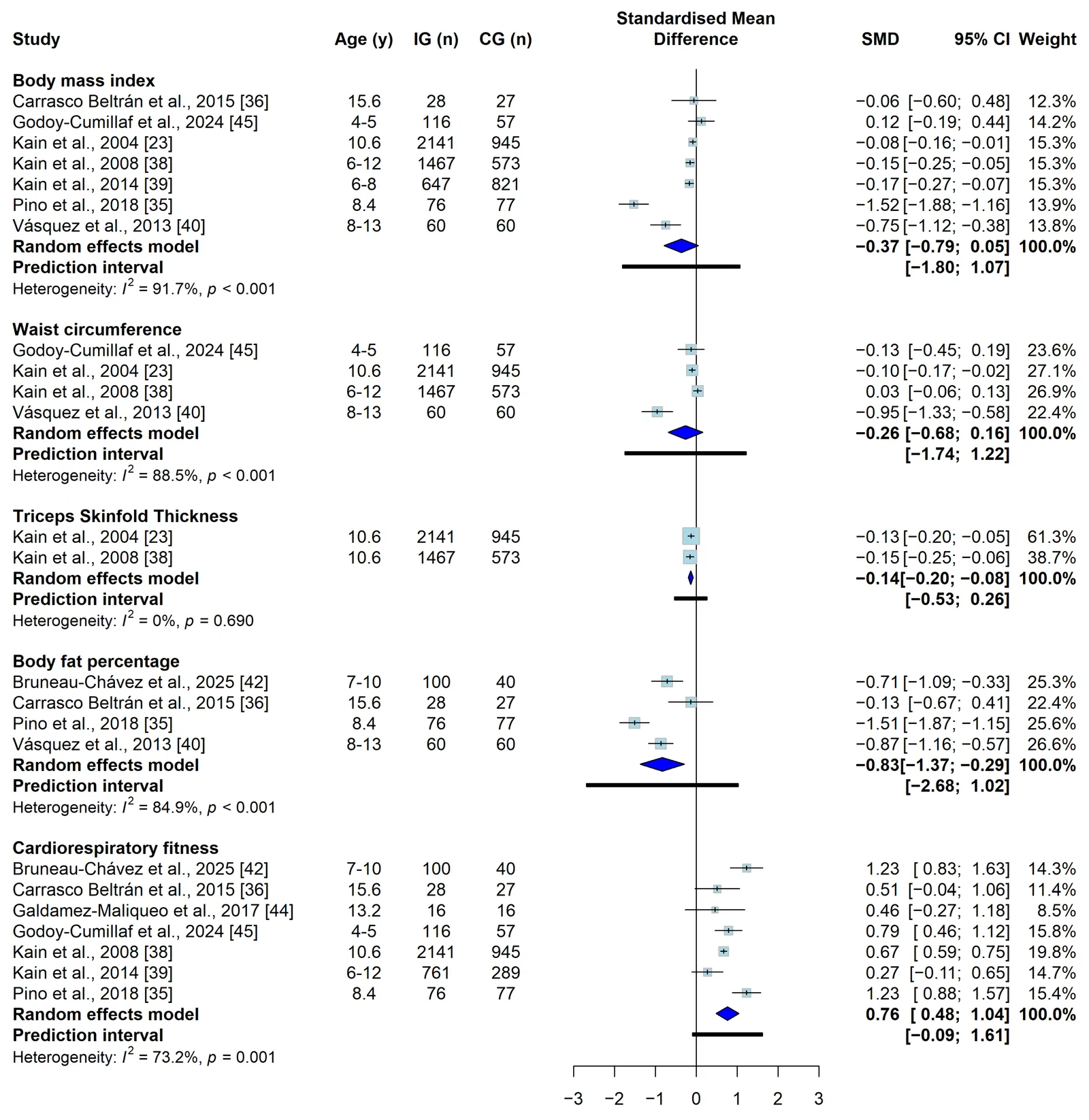
Meta-analyses of the effect of physical activity interventions on anthropometric outcomes and cardiorespiratory fitness in Chilean children and adolescents [23,35,36,38,39,40,42,44,45]. Squares represent individual study effect estimates, with size proportional to study weight; diamonds represent pooled random-effects estimates. Horizontal lines indicate 95% confidence intervals, and bold horizontal lines indicate 95% prediction intervals. CI, confidence interval; RCT, randomized controlled trial; SMD, standardized mean difference.

Overall, pooled estimates favored the physical activity interventions for triceps skinfold thickness, body fat percentage, and cardiorespiratory fitness, whereas no clear changes were observed in body mass index (BMI) or waist circumference (Figure 2).

### 3.7. Effect on Body Mass Index

Due to differences in study design among the included studies, the effects of physical activity interventions on BMI were evaluated using two separate meta-analyses. The first included studies with a concurrent control group, whereas the second included uncontrolled before–after studies, thereby allowing the available evidence to be considered while preserving methodological comparability.

**Figure 3:**
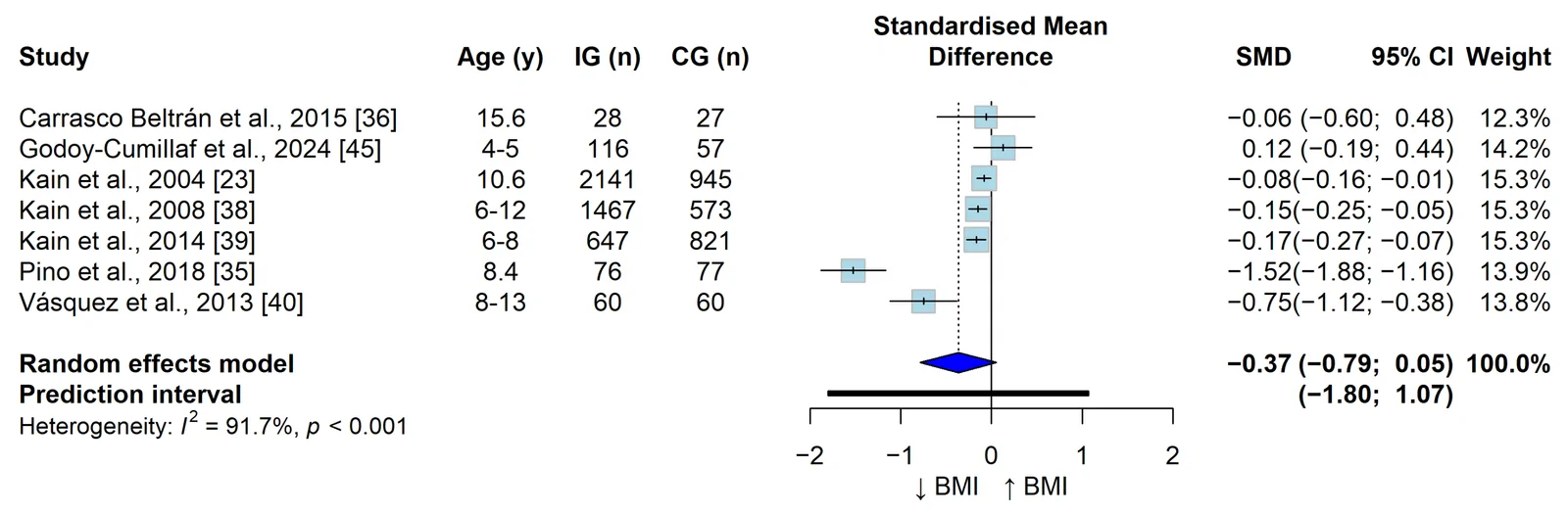
Effect of physical activity interventions on body mass index in studies with a concurrent control group [23,35,36,38,39,40,45]. Squares represent individual study effect estimates, with size proportional to study weight; diamonds represent pooled random-effects estimates. Horizontal lines indicate 95% confidence intervals, and bold horizontal lines indicate 95% prediction intervals. Arrows indicate the direction of the effect: negative SMDs (↓ BMI) indicate lower BMI in the intervention group relative to the control group, whereas positive SMDs (↑ BMI) indicate higher BMI. BMI, body mass index; CI, confidence interval; RCT, randomized controlled trial; SMD, standardized mean difference.

Seven studies with a concurrent control group contributed BMI data to quantitative synthesis. The pooled analysis showed a trend toward a reduction in BMI favoring the intervention group; however, the effect did not reach statistical significance (SMD = −0.37; 95% CI: −0.79 to 0.05; 95% prediction interval: −1.80 to 1.07). Between-study heterogeneity was considerable (I^2^ = 91.7%), indicating substantial variability across the included studies (Figure 3).

**Figure 4:**
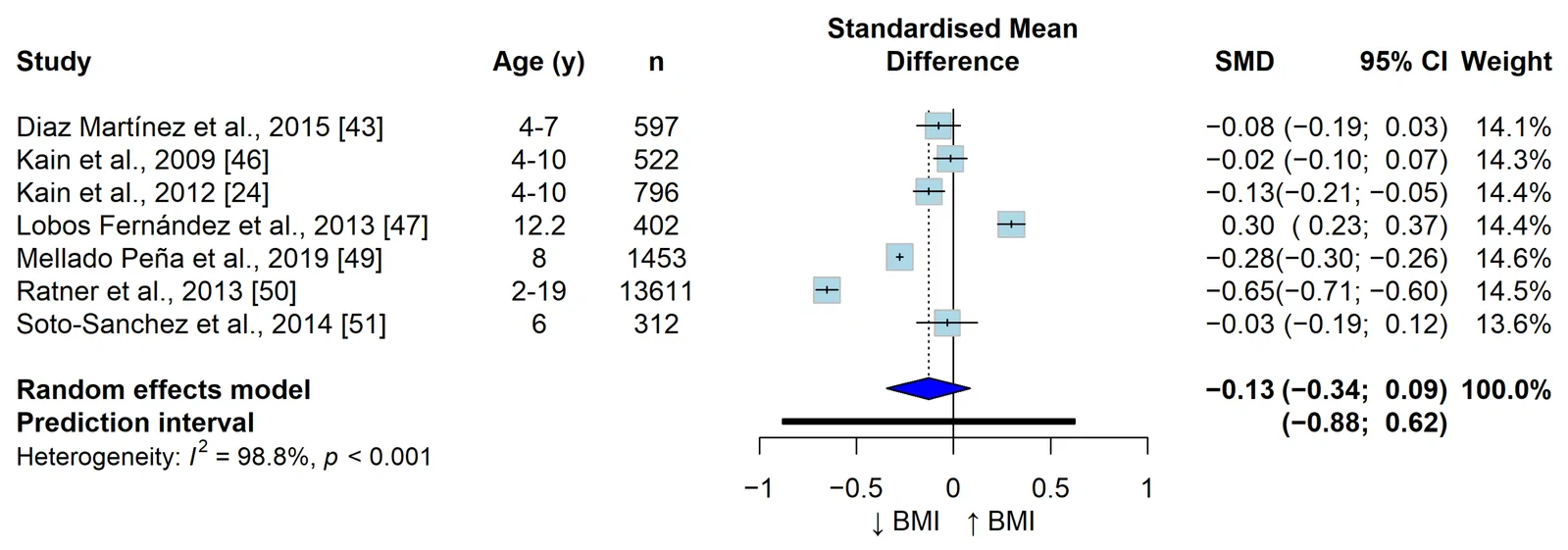
Effect of physical activity interventions on body mass index in uncontrolled before–after studies [24,43,46,47,49,50,51]. Squares represent individual study effect estimates, with size proportional to study weight; diamonds represent pooled random-effects estimates. Horizontal lines indicate 95% confidence intervals, and bold horizontal lines indicate 95% prediction intervals. Arrows indicate the direction of the effect: SMDs (↓ BMI) indicate lower BMI at post-intervention compared with baseline, whereas positive SMDs (↑ BMI) indicate higher BMI at post-intervention compared with baseline. BMI, body mass index; CI, confidence interval; SMD, standardized mean difference.

Similarly, seven uncontrolled before–after studies contributed to a separate meta-analysis of BMI. The pooled estimate suggested a small reduction in BMI from pre- to post-intervention; however, the effect was not statistically significant (SMD = −0.13; 95% CI: −0.34 to 0.09; 95% prediction interval: −0.88 to 0.62). Considerable heterogeneity was observed (I^2^ = 98.8%), reflecting substantial methodological and clinical variability across the included studies (Figure 4).

### 3.8. Effect on Waist Circumference

**Figure 5:**
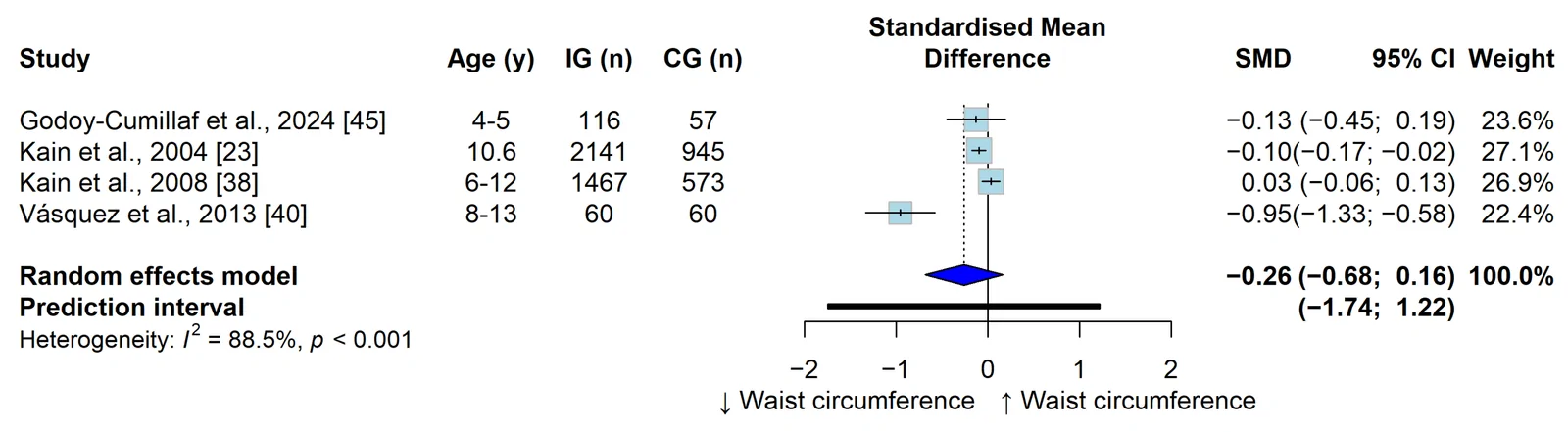
Effect of physical activity interventions on waist circumference [23,38,40,45]. Squares represent individual study effect estimates, with size proportional to study weight; diamonds represent pooled random-effects estimates. Horizontal lines indicate 95% confidence intervals, and bold horizontal lines indicate 95% prediction intervals. Arrows indicate the direction of the effect: negative SMDs (↓ Waist circumference) indicate lower waist circumference in the intervention group relative to the control group, whereas positive SMDs (↑ Waist circumference) indicate higher waist circumference. CI, confidence interval; RCT, randomized controlled trial; SMD, standardized mean difference.

Four studies evaluated the effects of physical activity interventions on waist circumference. The meta-analysis showed a non-significant reduction in waist circumference compared with the control groups (SMD = −0.05; 95% CI: −0.14 to 0.05; 95% prediction interval: −0.38 to 0.29). Considerable between-study heterogeneity was observed (I^2^ = 88.5%), indicating substantial variability across the included interventions (Figure 5).

### 3.9. Effect on Triceps Skinfold Thickness

**Figure 6:**
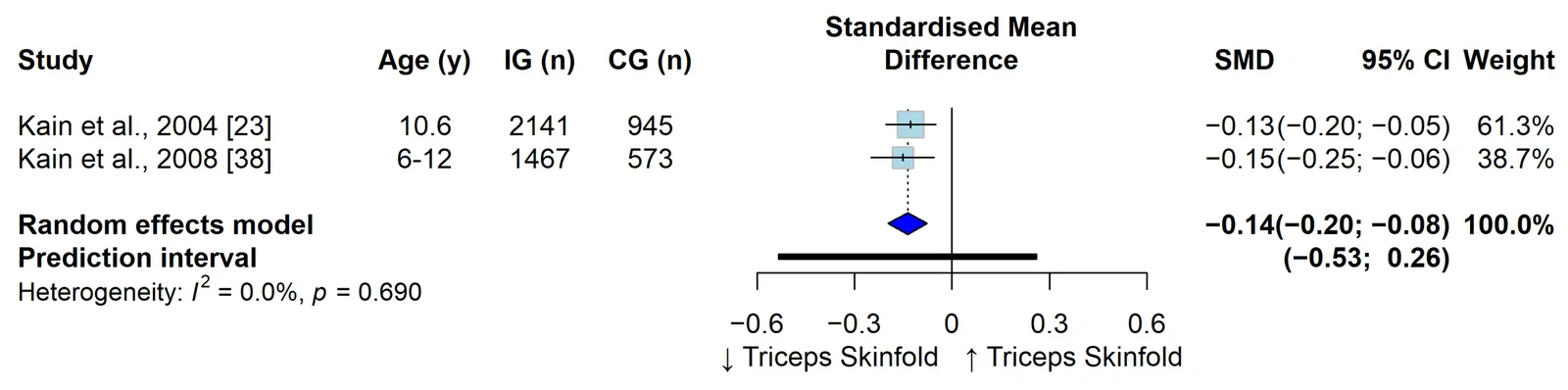
Effect of physical activity interventions on triceps skinfold thickness in studies with a concurrent control group [23,38]. Squares represent individual study effect estimates, with size proportional to study weight; diamonds represent pooled random-effects estimates. Horizontal lines indicate 95% confidence intervals, and bold horizontal lines indicate 95% prediction intervals. Arrows indicate the direction of the effect: negative SMDs (↓ Triceps skinfold) indicate lower triceps skinfoldthickness in the intervention group relative to the control group, whereas positive SMDs (↑ Triceps skinfold) indicate higher triceps skinfold thickness. CI, confidence interval; RCT, randomized controlled trial; SMD, standardized mean difference.

Two studies evaluated the effects of physical activity interventions on triceps skinfold thickness. The pooled estimate favored the intervention group (SMD = −0.14; 95% CI: −0.20 to −0.08; 95% prediction interval: −0.53 to 0.26), with no evidence of between-study heterogeneity (I^2^ = 0%). However, only two studies contributed to this analysis, and the 95% prediction interval included the null, limiting confidence in the consistency and reproducibility of the pooled estimate (Figure 6).

### 3.10. Effect on Body Fat Percentage

**Figure 7:**
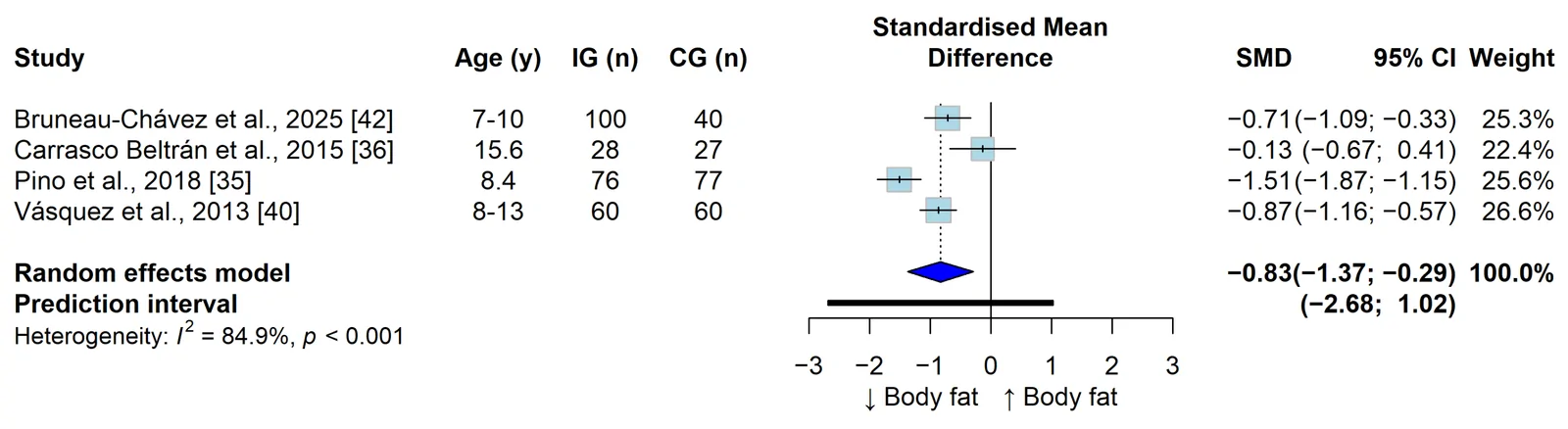
Effect of physical activity interventions on body fat percentage in studies with a concurrent control group [35,36,40,42]. Squares represent individual study effect estimates, with size proportional to study weight; diamonds represent pooled random-effects estimates. Horizontal lines indicate 95% confidence intervals, and bold horizontal lines indicate 95% prediction intervals. Arrows indicate the direction of the effect: negative SMDs (↓ Body fat) indicate lower body fat percentage in the intervention group relative to the control group, whereas positive SMDs (↑ Body fat) indicate higher body fat percentage. CI, confidence interval; RCT, randomized controlled trial; SMD, standardized mean difference.

Four studies evaluated the effects of physical activity interventions on body fat percentage. The pooled estimate favored the intervention group (SMD = −0.83; 95% CI: −1.37 to −0.29). However, considerable between-study heterogeneity was observed (I^2^ = 84.9%; *p* < 0.001), and the 95% prediction interval ranged from −2.68 to 1.02, indicating substantial uncertainty regarding the magnitude and reproducibility of the pooled association (Figure 7).

### 3.11. Effect on Cardiorespiratory Fitness

**Figure 8:**
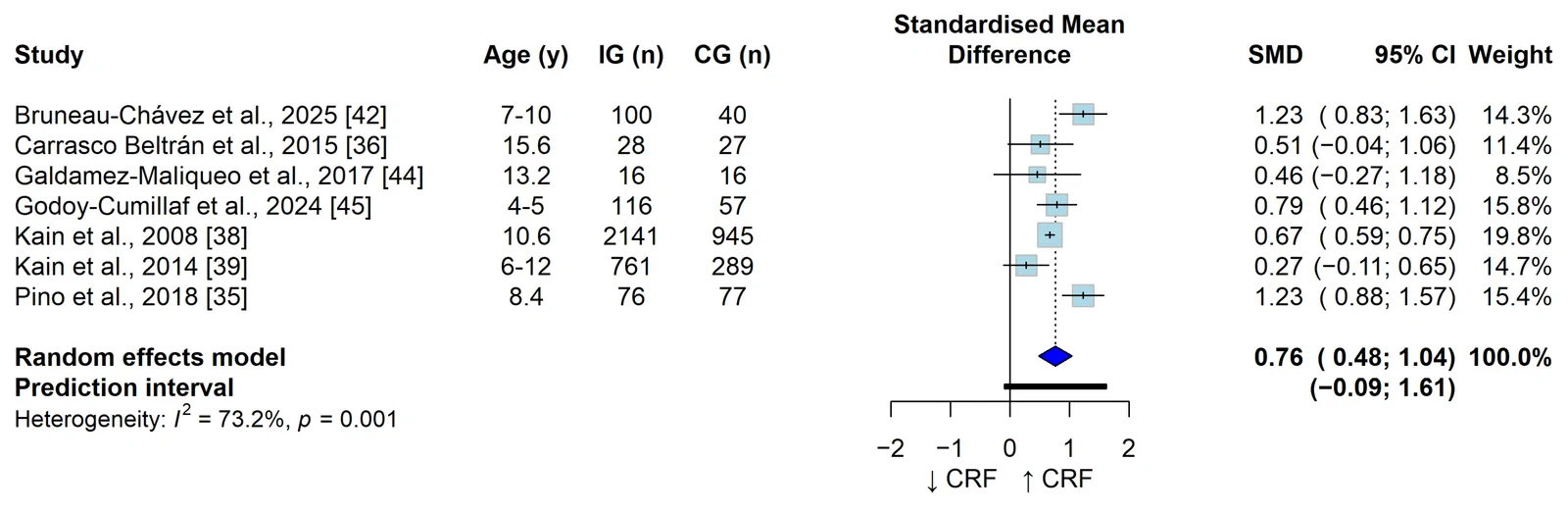
Effect of physical activity interventions on cardiorespiratory fitness in studies with a concurrent control group [35,36,38,39,42,44,45]. Squares represent individual study effect estimates, with size proportional to study weight; diamonds represent pooled random-effects estimates. Horizontal lines indicate 95% confidence intervals, and bold horizontal lines indicate 95% prediction intervals. Arrows indicate the direction of the effect: negative SMDs (↓ CRF) indicate lower cardiorespiratory fitness in the intervention group relative to the control group, whereas positive SMDs (↑ CRF) indicate higher cardiorespiratory fitness. CI, confidence interval; CRF, cardiorespiratory fitness; RCT, randomized controlled trial; SMD, standardized mean difference.

Seven studies contributed to the meta-analysis of cardiorespiratory fitness. The pooled estimate favored the intervention groups (SMD = 0.76; 95% CI: 0.48 to 1.04; 95% prediction interval: −0.09 to 1.61), corresponding to a moderate effect size. Substantial between-study heterogeneity was observed (I^2^ = 73.2%), indicating variability in the magnitude of the estimated association across intervention programs and assessment methods (Figure 8).

### 3.12. Sensitivity Analysis

Overall, the pooled effect estimates and between-study heterogeneity were not substantially altered when each study was excluded individually (Table S7). For uncontrolled before–after studies, sensitivity analyses using alternative assumed pre–post correlations yielded virtually identical pooled estimates (r = 0.30: SMD = −0.13, 95% CI −0.35 to 0.09; r = 0.70: SMD = −0.13, 95% CI −0.34 to 0.09), indicating that the findings were not sensitive to the assumed pre–post correlation (Table S8).

### 3.13. Risk of Bias Results

Risk of bias varied substantially according to study design.

The single cluster-randomized controlled trial was judged as having some concerns overall using the cluster-specific RoB 2 tool. Concerns primarily related to incomplete reporting of the randomization procedure and the timing of participant identification and recruitment, missing outcome data, and selection of the reported result, whereas deviations from the intended intervention and outcome measurement were judged at low risk.

**Figure 9:**
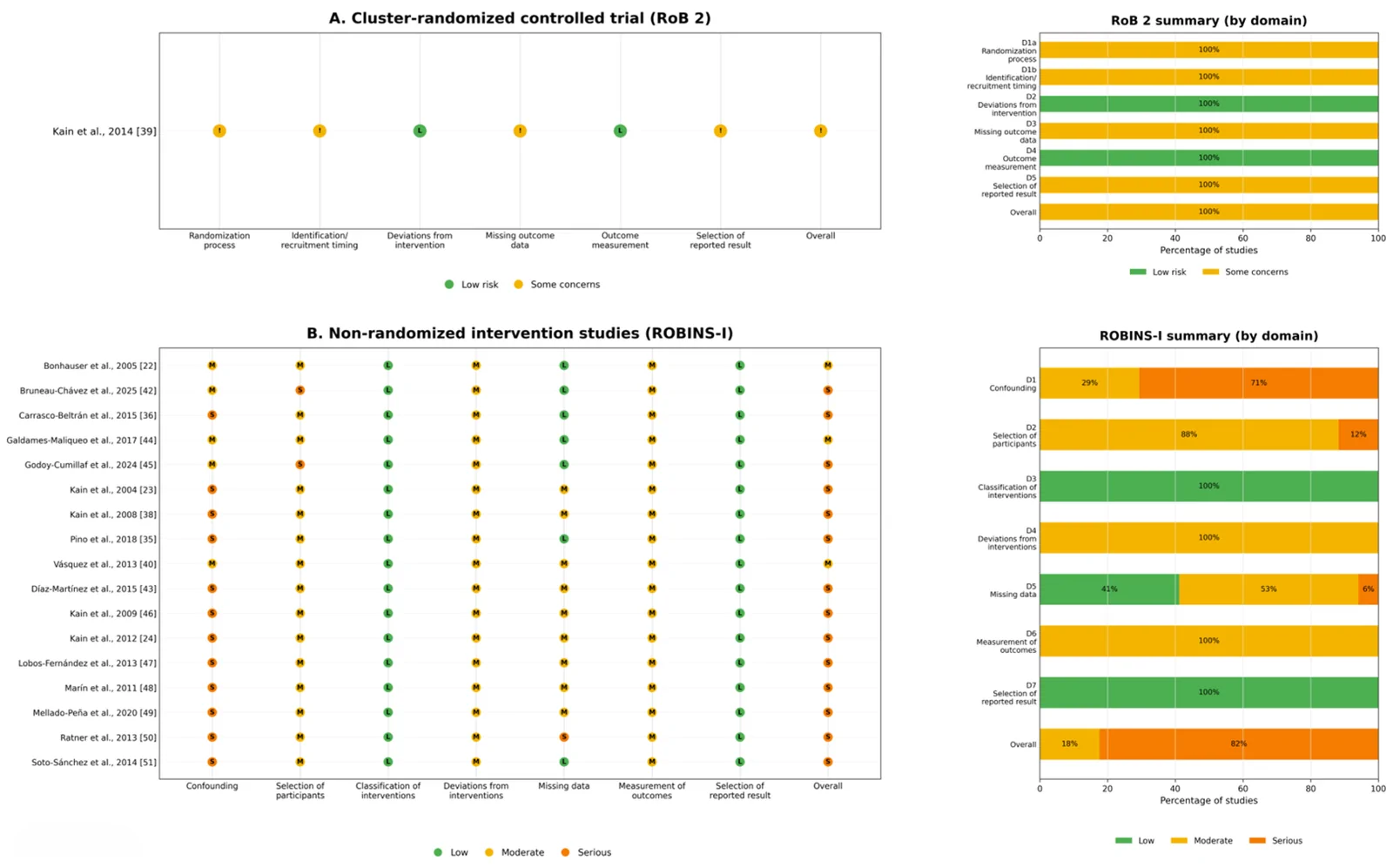
Risk of Bias Assessment of the Included Studies. (**A**) Risk-of-bias assessment of the cluster-randomized controlled trial using the cluster-specific RoB 2 tool [39]. (**B**) Risk-of-bias assessment of non-randomized controlled trials and uncontrolled before–after studies using ROBINS-I [22,23,24,35,36,38,40,42,43,44,45,46,47,48,49,50,51]. L, low rosk; M, moderate risk; S, serious risk; !, some concerns.

Among the 17 non-randomized intervention studies assessed using ROBINS-I, three (17.6%) were judged at moderate risk of bias and 14 (82.4%) at serious risk; none was judged at low or critical risk. Serious judgments were driven primarily by confounding. All eight uncontrolled before–after studies were judged at serious risk because, in the absence of a concurrent comparator, secular trends, maturation, regression to the mean, and other time-varying factors could not be distinguished from intervention effects. Additional serious concerns arose in several non-randomized controlled studies because of non-random allocation and potential baseline or contextual differences between groups, or post-intervention selection based on intervention adherence. Missing data represented an additional concern in some studies, particularly those with substantial attrition. Detailed domain-level judgments are presented in Figure 9.

### 3.14. Certainty of Evidence Results

The certainty of evidence was rated as very low for all six meta-analyzed outcomes, primarily because of very serious risk-of-bias concerns and serious to very serious inconsistency related to substantial or considerable between-study heterogeneity and uncertainty reflected by the prediction intervals (Table S4). Imprecision additionally affected the BMI and waist circumference estimates, while the evidence base was particularly limited for triceps skinfold thickness.

## 4. Discussion

To the best of our knowledge, this systematic review and meta-analysis represent the first synthesis of physical activity interventions addressing anthropometric, body composition, and physical fitness outcomes among Chilean children and adolescents. Pooled estimates favored the intervention conditions for cardiorespiratory fitness, body fat percentage, and triceps skinfold thickness, whereas no clear differences were observed for BMI or waist circumference. However, confidence in these findings is limited by the predominance of non-randomized designs, serious risk-of-bias concerns, substantial to considerable heterogeneity for several outcomes, prediction intervals that included the null for most outcomes, and very low certainty of evidence across all meta-analyzed outcomes. Accordingly, the pooled estimates should be interpreted cautiously and should not be regarded as definitive evidence of causal intervention effects.

Although formal subgroup analyses or meta-regression were not considered appropriate given the small number of studies contributing to each synthesis, several potential sources of between-study heterogeneity should be considered qualitatively. At the participant level, the included studies covered a wide age range, spanning different developmental stages, and differed in baseline anthropometric and fitness characteristics, which may influence responsiveness to physical activity interventions. At the intervention level, programs varied substantially in duration, frequency, intensity, and modality, ranging from HIIT and structured exercise to additional Physical Education, recreational games, and broader school-based physical activity programs. Twelve interventions were multicomponent and incorporated nutritional, educational, psychological, family, or environmental strategies, whereas six consisted exclusively of physical activity or exercise, potentially contributing to differences in observed effects. Comparator conditions also varied, including usual school curricula, traditional Physical Education, and no additional intervention, thereby affecting the contrast between intervention and comparison groups. Methodological heterogeneity was also relevant, as the evidence included a cluster-randomized trial, non-randomized controlled trials, and uncontrolled before–after studies, which differ in their susceptibility to confounding, maturation, secular trends, and other sources of bias. In addition, outcome assessment methods varied across studies, particularly for cardiorespiratory fitness and body composition, and school- or cluster-level implementation factors such as intervention fidelity, teacher involvement, adherence, and the effective dose delivered may also have differed across settings. Given the limited number of studies and incomplete reporting of several of these characteristics, their contribution to heterogeneity could not be formally quantified and should therefore be regarded as plausible sources of variability rather than established effect modifiers.

One of the main findings of the present meta-analysis was the absence of statistically significant BMI changes both in studies with a concurrent control group and in uncontrolled before–after studies. These findings are consistent with most interventions conducted in Chilean pediatric populations, as well as with the meta-analysis by García-Hermoso et al. [15], which reported small and clinically limited effects on BMI following physical activity interventions in schoolchildren, and with the reviews by Kelley and Kelley [6] and Muñoz-Urtubia et al. [52], which concluded that changes in this indicator tend to be modest when interventions do not include a nutritional component.

From a physiological perspective, the absence of significant changes in BMI does not necessarily indicate that the interventions were ineffective. During childhood and adolescence, linear growth, biological maturation, and developmental changes continuously modify body composition and body weight, potentially reducing the sensitivity of BMI to intervention-induced changes. Consequently, body weight may remain relatively stable despite meaningful improvements in body composition [8]. In this context, increases in muscle mass associated with regular physical activity may offset reductions in adipose tissue, thereby limiting the ability of BMI to reflect these adaptations [53]. This limitation has been widely documented and supports the recommendation to complement BMI with more specific indicators of adiposity and physical fitness [10]. Accordingly, incorporating complementary outcomes such as cardiorespiratory fitness and specific measures of adiposity may provide a more comprehensive assessment of intervention effects in children and adolescents.

From a clinical and public health perspective, these findings reinforce the need to avoid considering BMI as the sole indicator of intervention effectiveness in pediatric health promotion. Although BMI remains useful for epidemiological surveillance because of its simplicity and widespread use, its ability to detect changes induced by physical activity programs may be limited during periods of rapid growth [13]. Therefore, future interventions should incorporate complementary measures of adiposity and physical fitness to characterize the health benefits of physical activity in pediatric populations more accurately.

The present review found no statistically significant differences in waist circumference following physical activity interventions. Although the effect estimate favored the intervention group, the magnitude of the effect was small and did not reach statistical significance. This finding is consistent with systematic reviews conducted in pediatric populations [54,55,56], which have reported that school-based interventions relying primarily on physical activity generally produce modest or non-significant reductions in waist circumference, particularly when interventions are of limited duration or do not include complementary strategies targeting diet and other lifestyle behaviors [6,15,54].

Unlike BMI, waist circumference is a more specific indicator of central adiposity and has been closely associated with metabolic abnormalities and increased cardiovascular risk during childhood and adolescence [11,12]. However, abdominal fat accumulation is also influenced by growth, biological maturation, and participants’ baseline characteristics, which may attenuate the magnitude of changes observed following short- or medium-term interventions [10]. The limited number of studies and variation in the physical activity dose and co-intervention components may also have contributed to the uncertainty surrounding the pooled waist circumference estimate. Taken together, these findings do not rule out a potential beneficial effect of physical activity on central adiposity; rather, they suggest that the magnitude of this effect may depend on specific intervention and participant characteristics. Future studies should evaluate longer-duration programs with an adequate physical activity dose and longitudinal follow-up to determine more precisely their effects on this important marker of cardiometabolic risk [11].

In contrast to the findings for BMI and waist circumference, the present pooled estimate for triceps skinfold thickness favored the physical activity interventions. However, this finding was based on only two studies, the prediction interval included the null, and the certainty of evidence was very low. Therefore, the result should be regarded as suggestive rather than conclusive evidence of a reduction in subcutaneous adiposity even when broader anthropometric indicators do not show detectable changes [55]. This finding is consistent with evidence indicating that skinfold-derived measures may be more sensitive to changes in body fat during childhood and adolescence, particularly during periods of growth and development [16]. From a physiological perspective, this response may be explained by the greater specificity of skinfold thickness as a measure of subcutaneous adipose tissue compared with BMI. Whereas BMI reflects the relationship between body weight and height [10] without distinguishing between fat mass and fat-free mass, skinfold measurements can detect localized changes in body composition that may occur even when body weight remains relatively stable because of simultaneous increases in muscle mass associated with regular physical activity [57]. Therefore, if confirmed in future studies, reductions in triceps skinfold thickness could represent an adaptation that may not be captured by more general anthropometric indicators [11].

Nevertheless, these findings should be interpreted cautiously. Evidence for this outcome was based on only two studies, limiting the precision of the pooled estimate and precluding definitive conclusions regarding the magnitude and consistency of the effect. Differences in intervention characteristics, study populations, and measurement procedures may also have influenced the observed findings. Further research incorporating direct measures of body composition and a larger number of interventions is therefore required to determine whether reductions in triceps skinfold thickness represent a consistent effect of physical activity programs among Chilean pediatric populations.

The pooled estimate for cardiorespiratory fitness favored the intervention conditions. Although the magnitude of the pooled SMD was moderate, substantial heterogeneity and very low certainty of evidence limit confidence in the magnitude and causal interpretation of this association. In contrast to the anthropometric outcomes, this finding showed an overall favorable effect across all the included studies, suggesting that cardiorespiratory fitness may be particularly responsive to adaptations induced by physical activity programs implemented in school settings. This finding is consistent with international evidence showing that physical activity interventions can improve cardiorespiratory fitness during childhood and adolescence [58,59,60], even when changes in anthropometric indicators such as BMI or waist circumference are small or do not reach statistical significance [13,15]. From a physiological perspective, the observed improvements may reflect cardiovascular, respiratory, and muscular adaptations induced by regular exercise, including increased efficiency of oxygen transport and utilization, greater skeletal muscle oxidative capacity, and improved movement economy [60,61]. These adaptations may occur before detectable changes in body composition become apparent [13,14].

The clinical relevance of this finding extends beyond physical performance. Longitudinal studies have identified cardiorespiratory fitness as an important predictor of cardiovascular and metabolic health throughout childhood and adulthood [13,14], with higher levels being associated with a lower risk of obesity, metabolic syndrome, type 2 diabetes, and premature mortality. Accordingly, improving cardiorespiratory fitness represents an important target for interventions aimed at promoting pediatric health, even in the absence of substantial changes in body weight [15].

In this context, recent evidence [14,15] has proposed cardiorespiratory fitness as an important indicator for pediatric health surveillance because of its potential to identify cardiometabolic risk at an early stage and monitor the effects of interventions targeting children and adolescents. Together with the broader literature, these findings provide a rationale for including cardiorespiratory fitness as a complementary outcome in future school-based interventions and pediatric health surveillance, as it reflects relevant functional adaptations that may precede detectable changes in anthropometric indicators [13]. This is particularly relevant in the Chilean context, given the high prevalence of sedentary behavior, excess weight, and physical inactivity reported among children and adolescents [3,20]. From a public health perspective, school-based physical activity programs may contribute to improvements in cardiorespiratory fitness, an outcome closely associated with both current and future health benefits [2,3].

The present review has several strengths that support the methodological rigor, transparency, and reproducibility of the review. To the best of our knowledge, this is the first systematic review and meta-analysis to synthesize evidence on the effects of physical activity interventions implemented exclusively among Chilean children and adolescents using anthropometric, body composition, and physical fitness outcomes. Additional strengths include prospective protocol registration in PROSPERO, a comprehensive literature search across multiple databases, independent study selection and data extraction by reviewers, and systematic assessment of the risk of bias of the included studies following internationally recognized methodological guidance, including PRISMA and Cochrane recommendations. These features enhance the transparency, reproducibility, and methodological validity of the review.

Nevertheless, several limitations should be considered when interpreting the findings. First, the number of studies contributing to several outcome-specific meta-analyses was limited, particularly for triceps skinfold thickness, waist circumference, and body fat percentage, which reduced the precision of the pooled estimates and also precluded additional analyses such as meta-regressions to investigate potential effect modifiers (e.g., participant age) [28] and formal assessment of publication bias [61]. Second, substantial methodological and clinical heterogeneity was present across studies, including differences in intervention duration, frequency, intensity, program components, participant characteristics, and outcome assessment methods. Several interventions also had relatively short durations or follow-up periods, which may have limited the detection of changes in anthropometric outcomes. Third, a substantial proportion of the evidence came from non-randomized studies, including uncontrolled before–after designs. In these studies, pre–post changes cannot be attributed causally to the intervention because maturation, secular trends, regression to the mean, and other time-varying factors cannot be separated from intervention-related changes in the absence of a concurrent comparator. In addition, several studies evaluated multicomponent interventions; therefore, their observed effects cannot be attributed exclusively to physical activity. Incomplete or non-comparable reporting of nutritional, educational, psychological, and environmental components also precluded calculation of the relative contribution of physical activity to total intervention time in some studies. Furthermore, several studies included broad or overlapping age ranges and did not provide outcome data stratified by developmental stage, preventing reliable subgroup analyses comparing preschool children, school-aged children, and adolescents. Finally, the certainty of evidence was rated as very low for all meta-analyzed outcomes, primarily because of serious risk-of-bias concerns, inconsistency, and, for some outcomes, imprecision. Accordingly, confidence in the magnitude and causal interpretation of the pooled estimates is limited, and the findings should be interpreted with appropriate caution.

Future research should prioritize randomized controlled trials with larger sample sizes, longer intervention and follow-up periods, and more detailed reporting of intervention intensity and adherence. Further studies should also report anthropometric, body composition, and physical fitness outcomes using standardized procedures and provide age- or developmental-stage-stratified results to facilitate between-study comparisons and strengthen future quantitative syntheses. In addition, complementary measures of body composition (e.g., lean mass percentage) and physical fitness (e.g., speed-agility and flexibility), together with indicators of biological maturation (e.g., sexual maturation stage and age at peak height velocity), should be considered, as these factors may allow a more precise interpretation of the effects of physical activity interventions in children and adolescents.

## 5. Conclusions

Physical activity interventions implemented among Chilean children and adolescents were associated with improvements in cardiorespiratory fitness and reductions in triceps skinfold thickness and body fat percentage, whereas no clear differences were observed in BMI or waist circumference. However, the certainty of evidence was very low for all meta-analyzed outcomes, and substantial or considerable heterogeneity was present for several outcomes. Consequently, these findings should be regarded as uncertain and interpreted cautiously. Cardiorespiratory fitness and specific measures of adiposity and body composition may provide complementary information to general anthropometric indicators such as BMI, although whether they are consistently more responsive to physical activity interventions remains to be established. Well-designed randomized controlled trials with larger sample sizes, longer intervention and follow-up periods, standardized outcome reporting, and age- or developmental-stage-stratified analyses are needed to clarify these findings. This review provides an updated synthesis of the available evidence and may help guide the design and outcome assessment of future school-based physical activity research in Chile.

## Figures and Tables

**Table 1:** Characteristics of studies included in the systematic review and/or meta-analysis.

Reference	Country	Study Design	Age (Years)	Sample Size, n	Population	Intervention Type	Intervention Duration and Frequency	Physical Fitness Indicators	Physical Fitness Tests	Anthropometric and Body Composition Indicators
Bonhauser et al., 2005 [22]	Chile	Non-randomized controlled trial	15.5	198	Adolescents	PA-only, school-based PA program	90 min/session, 3 sessions/week, 1 academic year	CRF, speed, explosive lower-body strength	Yo-Yo Intermittent Recovery Test, 30-m speed test, countermovement jump	BMI
Bruneau-Chávez et al., 2025 [42]	Chile	Non-randomized controlled trial	7–10	140	Children	PA-only, HIIT incorporated into PE classes	45 min/session, 2 sessions/week, 12 weeks	CRF	20-m shuttle run	Body fat percentage (BIA)
Carrasco Beltrán, et al., 2015 [36]	Chile	Non-randomized controlled trial	15.6	55	Adolescents	PA-only, moderate-to-vigorous 3-vs.-3 small-sided games	60 min/session, 2 sessions/week, 11 weeks	CRF	20-m shuttle run	BMI, body fat percentage (BIA)
Díaz Martínez et al., 2015 [43]	Chile	Uncontrolled before–after study	6.1 (5–7)	306	Children	Multicomponent, school-based PA + healthy-eating education	PA 5 days/week, 5 months	Not applicable	Not applicable	BMI, waist circumference
Galdames-Maliqueo at al., 2017 [44]	Chile	Non-randomized controlled trial	13.3	32	Adolescents	PA-only, HIIT based on maximal aerobic speed	16 HIIT sessions	CRF	20-m shuttle run	BMI
Godoy-Cumillaf et al., 2024 [45]	Chile	Non-randomized controlled trial	4–5	173	Children	Multicomponent, ecological PA-promotion intervention	45 min/session, 3 sessions/week, 5 months	CRF, lower-body strength, speed-agility	Standing long jump, 4 × 10-m shuttle run, 20-m shuttle run	BMI, waist circumference
Kain et al., 2004 [22]	Chile	Non-randomized controlled trial	10.6 (6–12)	3086	Children	Multicomponent, school-based obesity-prevention intervention	Additional 90 min/week PA, 6 months	CRF, flexibility	20-m shuttle run, lower-back flexibility test	BMI, triceps skinfold thickness, waist circumference
Kain et al., 2008 [38]	Chile	Non-randomized controlled trial	9.9 (6–12)	2040	Children	Multicomponent, school-based obesity-prevention intervention	Additional 90 min/week PA, ~11 months	CRF	One-Mile Run Test, 20-m shuttle run	BMI, triceps skinfold thickness, waist circumference
Kain et al., 2009 [46]	Chile	Uncontrolled before–after study	4–10	522	Children	Multicomponent, school-based obesity-prevention pilot intervention	5 months; standardized PA dose not reported	CRF	6-min walk test	BMI, waist circumference
Kain et al., 2012 [24]	Chile	Uncontrolled before–after study	6.4 (4–7)	597	Children	Multicomponent, school-based obesity-prevention intervention	4 PE sessions/week, 3 years	CRF	6-min walk test	BMI, waist circumference
Kain et al., 2014 [39]	Chile	Cluster-randomized controlled trial	6.6 (6–8)	1468	Children	Multicomponent, school-based obesity-prevention intervention	2 PE classes/week, 12 months	Not applicable	Not applicable	BMI
Lobos Fernández et al., 2013 [47]	Chile	Uncontrolled before–after study	7.6 (4–11)	804	Children	Multicomponent, school-based nutrition + PA intervention	4 h PE/week, 2 years	CRF	6-min walk test	BMI
Marín et al., 2011 [48]	Chile	Uncontrolled before–after study	12.2 (6–18)	402	Children	Multicomponent obesity treatment program	60 min/session, 2 sessions/week, 16 sessions	Not applicable	Not applicable	BMI, waist circumference
Mellado Peña et al., 2019 [49]	Chile	Uncontrolled before–after study	2–19	13,611	Children	Multicomponent, Programa Vida Sana 2017	48–72 PA sessions over 6 months	CRF, lower-body strength	Standing broad jump, 6-min walk test, 30-s chair stand test	BMI
Pino et al., 2018 [35]	Chile	Non-randomized controlled trial	8.4 (7–10)	153	Children	PA-only, HIIT within PE classes	15 min/session, 2 sessions/week, 12 weeks	CRF	6-min walk test	BMI, body fat percentage (BIA)
Ratner et al., 2013 [50]	Chile	Uncontrolled before–after study	8.0 (7–9)	1453	Children	Multicomponent, school-based Vive Sano program	Additional 90 min/week PA, 2 years	Not applicable	Not applicable	BMI, waist circumference
Soto-Sanchez et al., 2014 [51]	Chile	Uncontrolled before–after study	7–15	156	Children	PA-only, dynamic recreational games during the longest school recess	45 min/session, 5 days/week, 12 weeks	Upper- and lower- body strength	Handgrip dynamometry, standing long jump	BMI, waist circumference
Vásquez et al., 2013 [40]	Chile	Non-randomized controlled trial	11.7 (8–13)	120	Children	Multicomponent, school-based muscular-strength exercise + nutrition education + psychological support	45 min/session, 3 sessions/week, 3 months	Not applicable	Not applicable	BMI, body fat percentage, waist circumference

Age is presented as mean and range, where available; otherwise, the available age statistic is reported. Detailed intervention components, including the dose of physical activity and non-physical-activity components, are provided in Table S6. BIA, bioelectrical impedance analysis; BMI, body mass index; CRF, cardiorespiratory fitness; HIIT, high-intensity interval training; PA, physical activity; PE, physical education.

## Data Availability

Data will be available upon reasonable request to the corresponding author.

## Supplementary Materials

The following supporting information can be downloaded at https://www.mdpi.com/article/10.3390/children13101368/s1. Table S1: PRISMA 2020 checklist; Table S2: Detailed search strategies by information source; Table S3: Outcome definitions, original metrics, direction of benefit, transformations, and effect contrasts; Table S4: Certainty-of-evidence assessment for meta-analyzed outcomes; Table S5: Studies assessed in full text and excluded, with reasons for exclusion; Table S6: Intervention components and relative contribution of physical activity in the included studies; Table S7: Leave-one-out sensitivity analyses; Table S8: Sensitivity analysis of BMI estimates using alternative pre–post correlations in uncontrolled before–after studies.

## Abbreviations

The following abbreviations are used in this manuscript:

BMI	Body Mass Index
CI	Confidence Interval
GRADE	Grading of Recommendations Assessment, Development and Evaluation
HIIT	High-Intensity Interval Training
PICOS	Population, Intervention, Comparison, Outcomes, and Study Design
PRISMA	Preferred Reporting Items for Systematic Reviews and Meta-Analyses
PROSPERO	International Prospective Register of Systematic Reviews
RoB2	Revised Cochrane Risk of Bias Tool for Randomized Trials
ROBINS-I	Risk of Bias In Non-randomized Studies of Interventions
SMD	Standardized Mean Difference
VO_2_max	Maximal Oxygen Uptake
