# Supplementary material for: Effects of Physical Activity Interventions on Anthropometric, Body Composition, and Physical Fitness Indicators in Chilean Children and Adolescents: Systematic Review and Meta-Analysis
Source: Children (Basel). 2026 Oct 8;13(10):1368. doi: 10.3390/children13101368 (PMC13703557; doi:10.3390/children13101368)
Supplement: Supplementary file 1 [file children-13-01368-s001.zip › children-4543380-supplementary.pdf]

SUPPLEMENTARY MATERIAL

Effects of Physical Activity Interventions on Anthropometric, Body Composition, and Physical Fitness Indicators in Chilean Children and Adolescents. Systematic Review and Meta-Analysis

INDEX

Table S1. PRISMA 2020 checklist.

Table S2. Detailed search strategies by information source.

Table S3. Outcome definitions, original metrics, direction of benefit, transformations, and effect contrasts.

Table S4. Certainty-of-evidence assessment for meta-analyzed outcomes.

Table S5. Studies assessed in full text and excluded, with reasons for exclusion.

Table S6. Intervention components and relative contribution of physical activity in the included studies.

Table S7. Leave-one-out sensitivity analyses.

Table S8. Sensitivity analysis of BMI estimates using alternative pre–post correlations in uncontrolled before–after studies

Table S1. PRISMA 2020 checklist.

Section and Topic	Item #	Checklist item	Location where item is reported
**TITLE**			
Title	1	Identify the report as a systematic review.	p.1
**ABSTRACT**			
Abstract	2	See the PRISMA 2020 for Abstracts checklist	pp. 1–2
**INTRODUCTION**			
Rationale	3	Describe the rationale for the review in the context of existing knowledge.	pp. 2–3
Objectives	4	Provide an explicit statement of the objective(s) or question(s) the review addresses.	p. 3
**METHODS**			
Eligibility criteria	5	Specify the inclusion and exclusion criteria for the review and how studies were grouped for the syntheses.	pp. 4–5; Sections 2.3–2.4; Table S3
Information sources	6	Specify all databases, registers, websites, organisations, reference lists and other sources searched or consulted to identify studies. Specify the date when each source was last searched or consulted.	p. 4; Section 2.2; Table S2
Search strategy	7	Present the full search strategies for all databases, registers and websites, including any filters and limits used.	p. 4; Section 2.2; Table S2
Selection process	8	Specify the methods used to decide whether a study met the inclusion criteria of the review, including how many reviewers screened each record and each report retrieved, whether they worked independently, and if applicable, details of automation tools used in the process.	pp. 4–5; Sections 2.1 and 2.5
Data collection process	9	Specify the methods used to collect data from reports, including how many reviewers collected data from each report, whether they worked independently, any processes for obtaining or confirming data from study investigators, and if applicable, details of automation tools used in the process.	pp. 4–5 and 7; Sections 2.1, 2.6, and 2.8.3
Data items	10a	List and define all outcomes for which data were sought. Specify whether all results that were compatible with each outcome domain in each study were sought (e.g. for all measures, time points, analyses), and if not, the methods used to decide which results to collect.	pp. 4–5; Sections 2.3 and 2.6; Table S3
	10b	List and define all other variables for which data were sought (e.g. participant and intervention characteristics, funding sources). Describe any assumptions made about any missing or unclear information.	p. 5; Section 2.6; Table 1; Table S6
Study risk of bias assessment	11	Specify the methods used to assess risk of bias in the included studies, including details of the tool(s) used, how many reviewers assessed each study and whether they worked independently, and if applicable, details of automation tools used in the process.	pp. 4–6; Sections 2.1 and 2.7
Effect measures	12	Specify for each outcome the effect measure(s) (e.g. risk ratio, mean difference) used in the synthesis or presentation of results.	p. 6; Section 2.8.1; Table S3
Synthesis methods	13a	Describe the processes used to decide which studies were eligible for each synthesis (e.g. tabulating the study intervention characteristics and comparing against the planned groups for each synthesis (item #5)).	pp. 6–7; Sections 2.8.1–2.8.2; Table S3
	13b	Describe any methods required to prepare the data for presentation or synthesis, such as handling of missing summary statistics, or data conversions.	pp. 5–7; Sections 2.6, 2.8.1, and 2.8.3;

Section and Topic	Item #	Checklist item	Location where item is reported
			Table S3
	13c	Describe any methods used to tabulate or visually display results of individual studies and syntheses.	pp. 6–7; Section 2.8.2
	13d	Describe any methods used to synthesize results and provide a rationale for the choice(s). If meta-analysis was performed, describe the model(s), method(s) to identify the presence and extent of statistical heterogeneity, and software package(s) used.	pp. 6–7; Section 2.8.2
	13e	Describe any methods used to explore possible causes of heterogeneity among study results (e.g. subgroup analysis, meta-regression).	Not performed because of the limited number of studies per outcome; discussed on p.20
	13f	Describe any sensitivity analyses conducted to assess robustness of the synthesized results.	p. 6; Section 2.8.2
Reporting bias assessment	14	Describe any methods used to assess risk of bias due to missing results in a synthesis (arising from reporting biases).	Not performed because of the limited number of studies per outcome; discussed on p.20
Certainty assessment	15	Describe any methods used to assess certainty (or confidence) in the body of evidence for an outcome.	p. 7; Section 2.9; Table S4
**RESULTS**			
Study selection	16a	Describe the results of the search and selection process, from the number of records identified in the search to the number of studies included in the review, ideally using a flow diagram.	pp. 7–8; Section 3.1; Figure 1
	16b	Cite studies that might appear to meet the inclusion criteria, but which were excluded, and explain why they were excluded.	p. 7; Section 3.1; Table S5
Study characteristics	17	Cite each included study and present its characteristics.	pp. 7–10; Sections 3.2–3.3; Table 1; Table S6
Risk of bias in studies	18	Present assessments of risk of bias for each included study.	pp. 16–17; Section 3.13; Figure 9
Results of individual studies	19	For all outcomes, present, for each study: (a) summary statistics for each group (where appropriate) and (b) an effect estimate and its precision (e.g. confidence/credible interval), ideally using structured tables or plots.	pp. 12–17; Sections 3.5–3.11; Figures 2–8
Results of syntheses	20a	For each synthesis, briefly summarise the characteristics and risk of bias among contributing studies.	pp. 7–12 and 18–20; Sections 3.2–3.4 and 3.13; Table 1; Figure 9; Table S4
	20b	Present results of all statistical syntheses conducted. If meta-analysis was done, present for each the summary estimate and its precision (e.g. confidence/credible interval) and measures of statistical heterogeneity. If comparing groups, describe the direction of the effect.	pp. 13–17; Sections 3.6–3.11; Figures 2–8

Section and Topic	Item #	Checklist item	Location where item is reported
	20c	Present results of all investigations of possible causes of heterogeneity among study results.	Not formally assessed because fewer than 10 studies contributed to each meta-analysis, discussed on p.20
	20d	Present results of all sensitivity analyses conducted to assess the robustness of the synthesized results.	p. 116; Section 3.12; Tables S7-S8
Reporting biases	21	Present assessments of risk of bias due to missing results (arising from reporting biases) for each synthesis assessed.	Not formally assessed because fewer than 10 studies contributed to each meta-analysis, discussed on p.20
Certainty of evidence	22	Present assessments of certainty (or confidence) in the body of evidence for each outcome assessed.	p. 17; Section 3.14; Table S4
**DISCUSSION**			
Discussion	23a	Provide a general interpretation of the results in the context of other evidence.	pp. 17–21; Section 4
	23b	Discuss any limitations of the evidence included in the review.	pp. 20-21
	23c	Discuss any limitations of the review processes used.	pp. 20-21
	23d	Discuss implications of the results for practice, policy, and future research.	p.21
**OTHER INFORMATION**			
Registration and protocol	24a	Provide registration information for the review, including register name and registration number, or state that the review was not registered.	p. 4; Section 2.1
	24b	Indicate where the review protocol can be accessed, or state that a protocol was not prepared.	p. 4; Section 2.1
	24c	Describe and explain any amendments to information provided at registration or in the protocol.	p. 4; Section 2.1, Table S1 [1]
Support	25	Describe sources of financial or non-financial support for the review, and the role of the funders or sponsors in the review.	p. 22
Competing interests	26	Declare any competing interests of review authors.	p. 22
Availability of data, code and other materials	27	Report which of the following are publicly available and where they can be found: template data collection forms; data extracted from included studies; data used for all analyses; analytic code; any other materials used in the review.	p. 22

[1] Amendments included expansion of the eligible age range from 4–18 to 2–19 years to encompass the pediatric age range represented in the eligible literature; inclusion of body composition and physical fitness outcomes; addition of Google Scholar and reference-list screening as supplementary search sources; use of SMDs instead of the prespecified MDs to accommodate conceptually comparable outcomes reported on different scales;

use of random-effects models throughout because methodological and clinical heterogeneity was anticipated; omission of subgroup analyses, meta-regression, and formal publication-bias assessment because fewer than 10 studies contributed to each outcome-specific meta-analysis; and addition of a certainty-of-evidence assessment using GRADE.

PRISMA 2020 for abstract checklist.

Section and Topic	Item #	Checklist item	Reported (Yes/No)
**TITLE**			
Title	1	Identify the report as a systematic review.	Yes
**BACKGROUND**			
Objectives	2	Provide an explicit statement of the main objective(s) or question(s) the review addresses.	Yes
**METHODS**			
Eligibility criteria	3	Specify the inclusion and exclusion criteria for the review.	No
Information sources	4	Specify the information sources (e.g. databases, registers) used to identify studies and the date when each was last searched.	Yes
Risk of bias	5	Specify the methods used to assess risk of bias in the included studies.	No
Synthesis of results	6	Specify the methods used to present and synthesise results.	Yes
**RESULTS**			
Included studies	7	Give the total number of included studies and participants and summarise relevant characteristics of studies.	Yes
Synthesis of results	8	Present results for main outcomes, preferably indicating the number of included studies and participants for each. If meta-analysis was done, report the summary estimate and confidence/credible interval. If comparing groups, indicate the direction of the effect (i.e. which group is favoured).	Yes
**DISCUSSION**			
Limitations of evidence	9	Provide a brief summary of the limitations of the evidence included in the review (e.g. study risk of bias, inconsistency and imprecision).	Yes
Interpretation	10	Provide a general interpretation of the results and important implications.	Yes
**OTHER**			
Funding	11	Specify the primary source of funding for the review.	No
Registration	12	Provide the register name and registration number.	Yes

Table S2. Detailed search strategies by information source.

Information source	Method
PubMed	("physical activity"[Title/Abstract] OR exercise[Title/Abstract] OR training[Title/Abstract] OR "exercise program"[Title/Abstract] OR "school-based intervention"[Title/Abstract] OR "physical education"[Title/Abstract]) AND (child*[Title/Abstract] OR adolescent*[Title/Abstract] OR youth[Title/Abstract] OR schoolchildren[Title/Abstract] OR preschool*[Title/Abstract]) AND (Chile[Title/Abstract] OR Chilean[Title/Abstract]) AND ("body mass index"[Title/Abstract] OR obesity[Title/Abstract] OR adiposity[Title/Abstract] OR anthropometric[Title/Abstract] OR "waist circumference"[Title/Abstract] OR skinfold[Title/Abstract] OR "physical fitness"[Title/Abstract] OR "cardiorespiratory fitness"[Title/Abstract] OR VO2max[Title/Abstract])<br>Fields: Title/Abstract. Filters/limits: no language, date, age, study-design, or publication-type filters.<br>Records retrieved: n = 134.
Scopus	TITLE-ABS-KEY(("physical activity" OR exercise OR "physical exercise" OR training OR "exercise program*" OR "physical activity intervention*" OR "exercise intervention*" OR "school-based intervention*" OR "physical education" OR "high intensity interval training" OR HIIT) AND (child* OR adolescent* OR youth OR schoolchild* OR preschool* OR pediatric* OR paediatric*) AND (Chile OR Chilean) AND ("body mass index" OR BMI OR anthropometr* OR adiposity OR "waist circumference" OR skinfold* OR "triceps skinfold" OR "tricipital skinfold" OR "physical fitness" OR "cardiorespiratory fitness" OR "aerobic fitness" OR "aerobic capacity" OR "cardiorespiratory endurance" OR VO2max OR "VO2 max" OR "VO2peak" OR "VO2 peak" OR "shuttle run" OR "20 m shuttle run"))<br>Fields: Title/Abstract/Keywords. Filters/limits: no language, date, age, study-design, or publication-type filters.<br>Records retrieved: n = 243.
Web of Science (All databases)	TS=(("physical activity" OR exercise OR "physical exercise" OR training OR "exercise program*" OR "physical activity intervention*" OR "exercise intervention*" OR "school-based intervention*" OR "physical education" OR "high intensity interval training" OR HIIT) AND (child* OR adolescent* OR youth OR schoolchild* OR preschool* OR pediatric* OR paediatric*) AND (Chile OR Chilean) AND ("body mass index" OR BMI OR anthropometr* OR adiposity OR "waist circumference" OR skinfold* OR "triceps skinfold" OR "tricipital skinfold" OR "physical fitness" OR "cardiorespiratory fitness" OR "aerobic fitness" OR "aerobic capacity" OR "cardiorespiratory endurance" OR VO2max OR "VO2 max" OR "VO2peak" OR "VO2 peak" OR "shuttle run" OR "20 m shuttle run"))<br>Fields: Topic (Title, Abstract, Author Keywords, and Keywords Plus). Filters/limits: no language, date, age, study-design, or publication-type filters.<br>Records retrieved: n = 164.
Google Scholar	Portal (URL): Google Scholar (https://scholar.google.com/).<br>Search terms: ("physical activity" OR exercise OR "physical education") AND (children OR adolescents) AND (Chile) AND ("body mass index" OR adiposity OR anthropometric OR "waist circumference" OR skinfold OR "physical fitness" OR "cardiorespiratory fitness" OR VO2max)<br>Procedure: results were screened in the default relevance-ranked order. The first 200 records were screened for eligibility. No language restriction was applied. Google Scholar does not provide a reproducible field-specific Title/Abstract filter equivalent to bibliographic databases; therefore, the search was run in the standard search interface using the same conceptual terms.
Citation searching	The reference lists of all included studies were manually screened to identify additional potentially eligible studies.

Notes: PubMed, Scopus, Web of Science, Google Scholar, and citation searching were last searched or consulted on 30 May 2026. Abbreviations: TITLE-ABS-KEY, title, abstract, keywords; TS, topic.

Table S3. Outcome definitions, original metrics, direction of benefit, transformations, and effect contrasts.

Outcome	Original metrics / units	Assessment / derivation in included studies	Direction of benefit	Transformations / harmonization for the review	Effect contrast used in synthesis	Synthesis status
BMI	kg/m^2; age- and sex-standardized BMI z-score (unitless)	BMI calculated from measured weight and height. Some studies reported raw BMI, whereas others reported BMI z-scores derived from pediatric growth references.	Lower values indicate benefit (reduced relative adiposity / lower BMI).	Continuous values were converted to standardized mean differences (SMDs), allowing raw BMI and BMI z-score metrics to be synthesized on a common standardized scale. No direct unit conversion was required. Effect direction was oriented so negative SMDs indicate benefit.	Controlled studies: between-group contrast in change from baseline, defined as Δ intervention – Δ control, where Δ = post − pre. Uncontrolled before–after studies: standardized mean change from pre- to post-intervention, accounting for the paired nature of the observations; r = 0.50 was assumed in the primary analysis, with r = 0.30 and r = 0.70 examined in sensitivity analyses.	Meta-analyzed separately for controlled and uncontrolled before–after studies.
Waist circumference	cm	Direct waist circumference measurement using standardized anthropometric procedures. Some source studies additionally classified elevated waist circumference using age-specific percentile cut-points, but the continuous measure was the relevant metric for synthesis.	Lower values indicate benefit (reduced central adiposity).	Converted to SMD. No unit conversion was required because studies reported waist circumference in centimeters. Effect direction was retained so negative SMDs indicate benefit.	Controlled studies: Δ intervention – Δ control, with Δ = post − pre.	Meta-analyzed in controlled studies.
Body fat percentage	% body fat	Percentage body fat was estimated primarily using bioelectrical impedance analysis (BIA).	Lower values indicate benefit (reduced adiposity).	Converted to SMD. No sign reversal was required because lower body fat percentage consistently represented benefit.	Controlled studies: Δ intervention – Δ control, with Δ = post − pre.	Meta-analyzed in controlled studies.
Triceps skinfold thickness	mm	Triceps skinfold thickness measured with skinfold calipers at the triceps site. In Kain et al. (2004), three measurements were averaged.	Lower values indicate benefit (reduced subcutaneous adiposity).	Converted to SMD and oriented so negative values indicate benefit. Kain et al. (2004) used natural-log-transformed triceps skinfold values for its own inferential analyses; the meta-analysis harmonized outcome estimates on the SMD scale.	Controlled studies: Δ intervention – Δ control, with Δ = post − pre.	Meta-analyzed in controlled studies.

Outcome	Original metrics / units	Assessment / derivation in included studies	Direction of benefit	Transformations / harmonization for the review	Effect contrast used in synthesis	Synthesis status
Cardiorespiratory fitness	VO_2max (mL·kg^{-1}·min^{-1}); 20-m shuttle-run stages; 6-minute walk distance (m); 6MWD/height ratio; one-mile run time/performance where reported	Field-based tests included the 20-m shuttle run / Course Navette, 6-minute walk test, and related estimates of maximal oxygen uptake. Some studies expressed fitness as distance normalized to height. One source study used 20-m shuttle-run stages as the score.	Higher VO_2max, more shuttle-run stages, greater walking distance, or a higher distance/height ratio indicate benefit. For any time-based endurance metric, a lower time indicates benefit.	Different scales were harmonized using SMDs. Direction was aligned so positive SMDs indicate improved cardiorespiratory fitness. Kain et al. (2004) used natural-log-transformed 20-m shuttle-run stage scores for its own inferential analysis; the meta-analysis synthesized standardized outcome estimates.	Controlled studies: Δ intervention – Δ control, with Δ = post − pre. Positive values were coded as favorable after direction harmonization.	Meta-analyzed in controlled studies; additional cardiorespiratory-fitness findings were summarized narratively when quantitative data were insufficient.
Muscular strength	Handgrip strength (kg); standing long-jump distance (cm); 30-s squat repetitions (n)	Upper-body strength was assessed using handgrip dynamometry; lower-limb performance using standing long jump; muscular endurance using repetitions completed in 30 s.	Higher strength, jump distance, or number of repetitions indicates benefit.	No pooled transformation was applied because these outcomes were not quantitatively synthesized. Results were retained in their original metrics for narrative reporting.	Controlled studies: intervention versus control change where available. Uncontrolled studies: post versus pre within the same participants.	Narrative synthesis only.
Speed-agility	Time (s)	Timed running/agility tests (e.g., 4 × 10 m speed-agility test; sprint-type tests in individual studies).	Lower completion time indicates benefit.	No pooled transformation was applied because this outcome was not quantitatively synthesized. If pooled in future analyses, the sign should be reversed so that a positive standardized effect consistently denotes improved fitness.	Controlled studies: intervention versus control change where available.	Narrative synthesis only.
Flexibility	Distance (cm)	Lower-back flexibility assessed as maximal reach distance from a standing position.	Greater reach distance indicates benefit.	No pooled transformation was applied because this outcome was not quantitatively synthesized; results were retained in centimeters.	Controlled study: intervention versus control change.	Narrative synthesis only.

Notes: Δ indicates change from baseline (post-intervention − pre-intervention). For anthropometric and adiposity outcomes, negative SMDs were coded as favorable; for fitness outcomes, positive SMDs were coded as favorable. Because SMDs were used, studies reporting conceptually comparable outcomes on different continuous scales could be combined without direct unit conversion. Abbreviations: BIA, bioelectrical impedance analysis; BMI, body mass index; 6MWD, six-minute walk distance; SMD, standardized mean difference; VO_2max, maximal oxygen uptake.

Table S4. Certainty-of-evidence assessment for meta-analyzed outcomes.

Research question: What is the effect of physical activity interventions on anthropometric, body-composition, and cardiorespiratory-fitness outcomes in Chilean children and adolescents?

Outcome	No. of studies	Study design	Risk of bias	Inconsistency	Indirectness	Imprecision	Other considerations	Effect (SMD, 95% CI; 95% PI)	Certainty	Plain-language summary
Body mass index – controlled studies	7	1 cluster-randomized controlled trial and 6 non-randomized controlled studies	Very serious[1]	Very serious[2]	Not serious	Serious[3]	Publication bias assessment: not applicable[7]	−0.37 (−0.79 to 0.05); PI −1.80 to 1.07	⊕◯◯◯ VERY LOW	The evidence is very uncertain about whether physical activity interventions reduce BMI compared with control conditions.
Body mass index – uncontrolled before–after studies	7	Uncontrolled before–after studies	Very serious[4]	Very serious[5]	Not serious	Serious[3]	Publication bias assessment: not applicable[7]	−0.13 (−0.34 to 0.09); PI −0.88 to 0.62	⊕◯◯◯ VERY LOW	The evidence is very uncertain about whether BMI changes after physical activity interventions in studies without a concurrent control group.
Waist circumference	4	Non-randomized controlled studies	Very serious[1]	Very serious[2]	Not serious	Serious[3]	Publication bias assessment: not applicable[7]	−0.05 (−0.14 to 0.05); PI −0.38 to 0.29	⊕◯◯◯ VERY LOW	The evidence is very uncertain; the pooled estimate suggests little or no average reduction in waist circumference.
Triceps skinfold thickness	2	Non-randomized controlled studies	Very serious[1]	Serious[6]	Not serious	Not serious	Publication bias assessment: not applicable[7]	−0.14 (−0.20 to −0.08); PI −0.53 to 0.26	⊕◯◯◯ VERY LOW	The pooled estimate suggests a small reduction, but the evidence is very uncertain because only two studies contributed and the prediction interval includes the null.
Body fat percentage	4	Non-randomized controlled studies	Very serious[1]	Very serious[2]	Not serious	Not serious	Publication bias assessment: not applicable[7]	−0.83 (−1.37 to −0.29); PI −2.68 to 1.02	⊕◯◯◯ VERY LOW	The pooled estimate suggests a reduction in body fat percentage, but the true effect is very uncertain because of serious risk of bias and considerable between-study inconsistency.
Cardiorespiratory fitness	7	Non-randomized controlled studies	Very serious[1]	Very serious[8]	Not serious	Not serious	Publication bias assessment: not applicable[7]	0.76 (0.48 to 1.04); PI −0.09 to 1.61	⊕◯◯◯ VERY LOW	The pooled estimate suggests improved cardiorespiratory fitness, but confidence in the magnitude and causal interpretation of the effect is very low.

Abbreviations: BMI, body mass index; CI, confidence interval; PI, prediction interval; SMD, standardized mean difference.

Notes: Certainty of evidence was assessed only for outcomes included in the quantitative meta-analysis. Because non-randomized studies were assessed using ROBINS-I, the ROBINS-I-linked GRADE approach was applied. Under this approach, the body of evidence was considered against a target randomized trial and was not automatically

downgraded solely because studies were non-randomized; instead, limitations related to non-randomization were incorporated within the risk-of-bias domain and downgraded according to their severity.

Explanations:

1. Downgraded two levels for risk of bias because the contributing controlled evidence was dominated by non-randomized studies, with most studies judged at serious overall risk of bias in the revised ROBINS-I assessment; the single cluster-randomized trial was judged as having some concerns.
2. Downgraded two levels for inconsistency because heterogeneity was considerable (BMI controlled: I^2 = 91.7%; waist circumference: I^2 = 88.5%; body fat percentage: I^2 = 84.9%) and the corresponding prediction intervals spanned materially different effects and included the null.
3. Downgraded one level for imprecision because the 95% confidence interval crossed the null and did not exclude both benefit and little/no effect (BMI controlled, BMI uncontrolled before–after, and waist circumference).
4. Downgraded two levels for risk of bias because all contributing studies used uncontrolled before–after designs. Without a concurrent comparator, secular trends, maturation, regression to the mean, and other time-varying factors cannot be separated from intervention-related change.
5. Downgraded two levels for inconsistency because heterogeneity was extreme (I^2 = 98.8%) and the 95% prediction interval ranged from −0.88 to 0.62, encompassing benefit, no effect, and possible worsening.
6. Downgraded one level for inconsistency because only two studies contributed. Although I^2 was 0% and the pooled 95% CI excluded the null, the 95% prediction interval (−0.53 to 0.26) included the null and effects in either direction; therefore, consistency cannot be established with confidence from such a sparse evidence base.
7. Publication bias was not formally assessed because fewer than 10 studies contributed to every meta-analysis. The inability to perform funnel-plot-based assessment was not, by itself, used as a reason to downgrade certainty.
8. Downgraded two levels for inconsistency because heterogeneity was substantial (I^2 = 73.2%) and the 95% prediction interval included null (−0.09 to 1.61).

Table S5. Studies assessed in full text and excluded, with reasons for exclusion.

Reason for exclusion	n
Ineligible publication type (e.g., reviews)	6
Ineligible population (non-Chilean population and/or adults)	17
Ineligible outcome (did not report an eligible anthropometric, body composition, or physical fitness outcome)	5
Ineligible study design (e.g., observational study without an eligible intervention or pre–post evaluation)	12
Total	40

Ineligible publication/study type (n = 6)

1. Al-Khudairy L, Loveman E, Colquitt JL, Mead E, Johnson RE, Fraser H, Olajide J, Murphy M, Velho RM, O'Malley C, Azevedo LB, Ells LJ, Metzendorf MI, Rees K. Diet, physical activity and behavioural interventions for the treatment of overweight or obese adolescents aged 12 to 17 years. Cochrane Database Syst Rev. 2017 Jun 22;6(6):CD012691. doi: 10.1002/14651858.CD012691.
2. Brown T, Moore TH, Hooper L, Gao Y, Zayegh A, Ijaz S, Elwenspoek M, Foxen SC, Magee L, O'Malley C, Waters E, Summerbell CD. Interventions for preventing obesity in children. Cochrane Database Syst Rev. 2019 Jul 23;7(7):CD001871. doi: 10.1002/14651858.CD001871.
3. Friedrich RR, Schuch I, Wagner MB. Effect of interventions on the body mass index of school-age students. Rev Saude Publica. 2012 Jun;46(3):551-60. English, Portuguese. doi: 10.1590/s0034-89102012005000036.
4. Lobelo F, Garcia de Quevedo I, Holub CK, Nagle BJ, Arredondo EM, Barquera S, Elder JP. School-based programs aimed at the prevention and treatment of obesity: evidence-based interventions for youth in Latin America. J Sch Health. 2013 Sep;83(9):668-77. doi: 10.1111/josh.12080.
5. Mead E, Brown T, Rees K, Azevedo LB, Whittaker V, Jones D, Olajide J, Mainardi GM, Corpeleijn E, O'Malley C, Beardsmore E, Al-Khudairy L, Baur L, Metzendorf MI, Demaio A, Ells LJ. Diet, physical activity and behavioural interventions for the treatment of overweight or obese children from the age of 6 to 11 years. Cochrane Database Syst Rev. 2017 Jun 22;6(6):CD012651. doi: 10.1002/14651858.CD012651.
6. Rocha Silva D., Martín-Matillas M., Carbonell-Baeza A., Aparicio V. A., Delgado-Fernández M. Efectos de los programas de intervención enfocados al tratamiento del sobrepeso/obesidad infantil y adolescente. Rev Andal Med Deporte [Internet]. 2014 Mar [citado 2026 Ago 18];7(1):33-43. doi:S1888-75462014000100006es.

Ineligible population (non-Chilean population and/or adults) (n = 17)

7. Alvarez C, Ramírez R, Flores M, Zúñiga C, Celis-Morales CA. Efectos del ejercicio físico de alta intensidad y sobrecarga en parámetros de salud metabólica en mujeres sedentarias, pre-diabéticas con sobrepeso u obesidad [Effect of sprint interval training and resistance exercise on metabolic markers in overweight women]. Rev Med Chil. 2012 Oct;140(10):1289-96. Spanish. doi: 10.4067/S0034-98872012001000008. PMID: 23559286.
8. Alvarez C, Ramirez-Campillo R, Martinez-Salazar C, Mancilla R, Flores-Opazo M, Cano-Montoya J, Ciolac EG. Low-Volume High-Intensity Interval Training as a Therapy for Type 2 Diabetes. Int J Sports Med. 2016 Aug;37(9):723-9. doi: 10.1055/s-0042-104935.

9. Alvirde-García Ulices, Rodríguez-Guerrero Alfredo J, Henao-Morán Santiago, Gómez-Pérez Francisco J, Aguilar-Salinas Carlos A. Resultados de un programa comunitario de intervención en el estilo de vida en niños. Salud pública Méx [revista en la Internet]. 2013 [citado 2026 Ago 18];55(Suppl 3):406-414. Disponible en: http://www.scielo.org.mx/scielo.php?script=sci_arttext&pid=S0036-36342013000900007&lng=es.
10. Andrade DC, Flores-Opazo M, Peñailillo L, Delgado-Floody P, Cano-Montoya J, Vásquez-Gómez JA, Alvarez C. Similar Adaptations to 10 Weeks Concurrent Training on Metabolic Markers and Physical Performance in Young, Adult, and Older Adult Women. J Clin Med. 2021 Nov 27;10(23):5582. doi: 10.3390/jcm10235582.
11. Dâmaso AR, Tock L, Tufik S, et al. Tratamento multidisciplinar reduz o tecido adiposo visceral, leptina, grelina e a prevalência de esteatose hepática não alcoólica (NAFLD) em adolescentes obesos. Rev Bras Med Esporte. 2006;12(5):263-267. DOI: 10.1590/S1517-86922006000500008.
12. Delgado Floody P, Jerez Mayorga D, Caamaño Navarrete F, Osorio Poblete A, Thuillier Lepeley N, Alarcón Hormazábal M. doce semanas de ejercicio físico intervalado con sobrecarga mejora las variables antropométricas de obesos mórbidos y obesos con comorbilidades postulantes a cirugía bariátrica [twelve weeks of physical exercise interval with surcharge improves the anthropometric variables of obese morbid and obese with comorbidities candidates to bariatric surgery]. Nutr Hosp. 2015 Nov 1;32(5):2007-11. Spanish. doi: 10.3305/nh.2015.32.5.9610.
13. Eisenmann JC, Laurson KR, Wickel EE, Gentile D, Walsh D. Utility of pedometer step recommendations for predicting overweight in children. Int J Obes (Lond). 2007 Jul;31(7):1179-82. doi: 10.1038/sj.ijo.0803553.
14. Farias ES, Paula F, Carvalho WRG, Gonçalves EM, Baldin AD, Guerra-Júnior G. Efeito da atividade física programada sobre a composição corporal em escolares adolescentes. J Pediatr (Rio J). 2009;85:28-34.
15. Fernández-Sánchez A, Sánchez-López M, Ruiz-Hermosa A, Redondo-Tébar A, Pozuelo-Carrascosa DP, Jiménez-López E, Martínez-Vizcaíno V. Effectiveness of a High-Intensity Interval Games Program in Extra-Curricular Context (MOVI-daFIT!) on Gross Motor Competence and Health-Related Quality of Life in Primary Schoolchildren: Cluster-Randomized Trial. Eur J Sport Sci. 2025 Jun;25(6):e12308. doi: 10.1002/ejsc.12308.
16. Fuentes LL, Muñoz AA. Efectos de una intervención integral en la reducción de factores de riesgo cardiovascular en mujeres con sobrepeso u obesidad de la Región de Los Ríos [Results of an integral lifestyle modification program to reduce weight among overweight and obese women]. Rev Med Chil. 2010 Aug;138(8):974-81. Spanish. Epub 2010 Nov 26. PMID: 21140054.
17. Mora-Gonzalez J, Esteban-Cornejo I, Solis-Urra P, Rodriguez-Ayllon M, Cadenas-Sanchez C, Hillman CH, Kramer AF, Catena A, Ortega FB. The effects of an exercise intervention on neuroelectric activity and executive function in children with overweight/obesity: The ActiveBrains randomized controlled trial. Scand J Med Sci Sports. 2024 Jan;34(1):e14486. doi: 10.1111/sms.14486.
18. Mujica V, Urzúa A, Leiva E, Díaz N, Moore-Carrasco R, Vásquez M, Rojas E, Icaza G, Toro C, Orrego R, Palomo I. Intervention with education and exercise reverses the metabolic syndrome in adults. J Am Soc Hypertens. 2010 May-Jun;4(3):148-53. doi: 10.1016/j.jash.2010.02.008.
19. Ortega FB, Mora-Gonzalez J, Cadenas-Sanchez C, Esteban-Cornejo I, Migueles JH, Solis-Urra P, Verdejo-Román J, Rodriguez-Ayllon M, Molina-Garcia P, Ruiz JR, Martinez-Vizcaino V, Hillman CH, Erickson KI, Kramer AF, Labayen I, Catena A. Effects of an Exercise Program on Brain Health Outcomes for Children With Overweight or Obesity: The

ActiveBrains Randomized Clinical Trial. JAMA Netw Open. 2022 Aug 1;5(8):e2227893. doi: 10.1001/jamanetworkopen.2022.27893.

20. Patiño Villada Fredy Alonso, Márquez Arabia Jorge Jaime, Uscátegui Peñuela Rosa Magdalena, Estrada Restrepo Alejandro, Agudelo Ochoa Gloria María, Manjarrés Luz Mariela et al. Efecto de una intervención con ejercicio físico y orientación nutricional sobre componentes del síndrome metabólico en jóvenes con exceso de peso. Iatreia [Internet]. 2013 Jan [cited 2026 Aug 18];26(1):34-43. Available from: http://www.scielo.org.co/scielo.php?script=sci_arttext&pid=S0121-07932013000100004&lng=en.
21. Prado DM, Silva AG, Trombetta IC, Ribeiro MM, Nicolau CM, Guazzelli IC, Matos LN, Negrao CE, Villares SM. Weight loss associated with exercise training restores ventilatory efficiency in obese children. Int J Sports Med. 2009 Nov;30(11):821-6. doi: 10.1055/s-0029-1233486.
22. Redondo-Tebar A, Ruiz-Hermosa A, Martinez-Vizcaino V, Bermejo-Cantarero A, Cavero-Redondo I, Martín-Espinosa NM, Sanchez-Lopez M. Effectiveness of MOVI-KIDS programme on health-related quality of life in children: Cluster-randomized controlled trial. Scand J Med Sci Sports. 2023 May;33(5):660-669. doi: 10.1111/sms.14291.
23. Sánchez-López M, Cavero-Redondo I, Álvarez-Bueno C, Ruiz-Hermosa A, Pozuelo-Carrascosa DP, Díez-Fernández A, Gutierrez-Díaz Del Campo D, Pardo-Guijarro MJ, Martínez-Vizcaíno V. Impact of a multicomponent physical activity intervention on cognitive performance: The MOVI-KIDS study. Scand J Med Sci Sports. 2019 May;29(5):766-775. doi: 10.1111/sms.13383.

Ineligible outcome (did not report an eligible anthropometric, body composition, or physical fitness outcome) (n = 5)

24. Cortinez-O'Ryan A, Albagli A, Sadarangani KP, Aguilar-Farias N. Reclaiming streets for outdoor play: A process and impact evaluation of "Juega en tu Barrio" (Play in your Neighborhood), an intervention to increase physical activity and opportunities for play. PLoS One. 2017 Jul 3;12(7):e0180172. doi: 10.1371/journal.pone.0180172.
25. Hormazábal-Aguayo I, Fernández-Vergara O, González-Calderón N, Vicencio-Rojas F, Russell-Guzmán J, Chacana-Cañas C, del Pozo-Cruz B, García-Hermoso A. Can a before-school physical activity program decrease bullying victimization in disadvantaged children? The Active-Start Study. Int J Clin Health Psychol. 2019;19(3):237–242. doi:10.1016/j.ijchp.2019.05.001.
26. Reyes-Amigo T, Grao-Cruces A, Sanchez-Oliva D, Garcia-Hermoso A, Reyes-Molina D, Yañez-Sepúlveda R, Olivares-Arancibia J, Hurtado-Almonácid J, Páez-Herrera J, Salinas-Gallardo G, Mendoza E, Ovalle-Fernández C, Sepúlveda-Figueroa F, Ibarra-Mora J. Acute effect of school-based active breaks on physical activity level and on-task classroom behavior in primary schoolchildren. Front Public Health. 2025 Oct 30;13:1644819. doi: 10.3389/fpubh.2025.1644819.
27. Robles-Campos A, Reyes-Molina D, Kracht-Suazo K, Cigarroa I, Cárcamo-Oyarzun J, Martinez-Lopez N, Perez-Ruiz M, Grao-Cruces A, Mota J, Ruiz-Ariza A, Muñoz Hinrichsen F, García-Pérez-de-Sevilla G, Celis-Morales C, Zapata-Lamana R. Effects of Video-Guided Active Breaks on Motor Competence of Schoolchildren with Special Education Needs. Children (Basel). 2025 Jun 21;12(7):820. doi: 10.3390/children12070820.
28. Rodríguez-Rodríguez F, Cristi-Montero C, Castro-Piñero J. Physical Activity Levels of Chilean Children in a National School Intervention Programme. A Quasi-Experimental Study. Int J Environ Res Public Health. 2020 Jun 23;17(12):4529. doi: 10.3390/ijerph17124529.

Ineligible study design (no eligible intervention comparison or pre–post evaluation) (n = 12)

29. Avalos C, Díaz C, Martínez A, Bancalari R, Zamorano J, Harbin F, Cerda V, Fernández M, Cavada G, Arteaga J, Valenzuela M, Toro M, García H. Waist circumference percentiles in children and adolescents between 6 and 14 years from Santiago, Chile. Endocrinol Nutr. 2012 May;59(5):296-303. doi: 10.1016/j.endonu.2012.02.007.
30. Burrows R, Correa-Burrows P, Reyes M, Blanco E, Albala C, Gahagan S. High cardiometabolic risk in healthy Chilean adolescents: associations with anthropometric, biological and lifestyle factors. Public Health Nutr. 2016 Feb;19(3):486-93. doi: 10.1017/S1368980015001585.
31. Burrows R, Correa-Burrows P, Reyes M, Blanco E, Albala C, Gahagan S. Low muscle mass is associated with cardiometabolic risk regardless of nutritional status in adolescents: A cross-sectional study in a Chilean birth cohort. Pediatr Diabetes. 2017 Dec;18(8):895-902. doi: 10.1111/pedi.12505.
32. Bustos-Barahona R, Delgado-Floody P, Martínez-Salazar C. Lifestyle associated with physical fitness related to health and cardiometabolic risk factors in Chilean schoolchildren. Endocrinol Diabetes Nutr (Engl Ed). 2020 67(9):586-593.doi: 10.1016/j.endinu.2020.02.005.
33. Delgado Floody PA, Caamaño Navarrete F, Palomino-Devia C, Jerez-Mayorga D, Martínez-Salazar C. Relationship in obese Chilean schoolchildren between physical fitness, physical activity levels and cardiovascular risk factors. Nutr Hosp. 2019;36(1):13-19. doi: 10.20960/nh.1932.
34. Ferrari G, Cofre Bolados C, Suárez-Reyes M, Farias Valenzuela C, Drenowatz C, Marques A, Pizarro T. Association of physical activity, muscular strength, and obesity indicators with self-concept in Chilean children. Nutr Hosp. 2022 Oct 17;39(5):1004-1011. English. doi: 10.20960/nh.04061.
35. García-Hermoso A, Agostinis-Sobrinho C, Camargo-Villalba GE, González-Jiménez NM, Izquierdo M, Correa-Bautista JE, Ramírez-Vélez R. Normal-Weight Obesity Is Associated with Poorer Cardiometabolic Profile and Lower Physical Fitness Levels in Children and Adolescents. Nutrients. 2020;12(4):1171. doi: 10.3390/nu12041171.
36. García-Rubio J, Olivares PR, Lopez-Legarrea P, Gómez-Campos R, Cossio-Bolaños MA, Merellano-Navarro E. asociación entre la calidad de vida relacionada con la salud, el estado nutricional (imc) y los niveles de actividad física y condición física en adolescentes chilenos [association between health related quality of life, bodyweight status (bmi) and physical activity and fitness levels in chilean adolescents]. Nutr Hosp. 2015 Oct 1;32(4):1695-702. doi: 10.3305/nh.2015.32.4.9182.
37. Godoy-Cumillaf A, Fuentes-Merino P, Farías-Valenzuela C, Duclos-Bastías D, Giakoni-Ramírez F, Bruneau-Chávez J, Merellano-Navarro E. The Association between Sedentary Behavior, Physical Activity, and Physical Fitness with Body Mass Index and Sleep Time in Chilean Girls and Boys: A Cross-Sectional Study. Children (Basel). 2023 May 31;10(6):981. doi: 10.3390/children10060981.
38. Gómez-Campos R, Andruske CL, Hespanhol J, Torres JS, Arruda M, Luarte-Rocha C, Cossio-Bolaños MA. Waist Circumferences of Chilean Students: Comparison of the CDC-2012 Standard and Proposed Percentile Curves. Int J Environ Res Public Health. 2015 Jul 9;12(7):7712-24. doi: 10.3390/ijerph120707712.
39. Palacio-Agüero A, Díaz-Torrente X, Quintiliano Scarpelli Dourado D. Relative handgrip strength, nutritional status and abdominal obesity in Chilean adolescents. PLoS One. 2020 Jun 10;15(6):e0234316. doi: 10.1371/journal.pone.0234316.

40. Zurita-Ortega F, Castro-Sánchez M, Rodríguez-Fernández S, Cofré-Boladós C, Chacón-Cuberos R, Martínez-Martínez A, Muros-Molina JJ. Actividad física, obesidad y autoestima en escolares chilenos: Análisis mediante ecuaciones estructurales [Physical activity, obesity and self-esteem in chilean schoolchildren]. Rev Med Chil. 2017 Mar;145(3):299-308. Spanish. doi: 10.4067/S0034-98872017000300006.

Table S6. Intervention components and relative contribution of physical activity in the included studies.

Study	Intervention classification	Physical activity component	Physical activity dose	Other intervention component(s)	Dose of other component(s)	PA share of scheduled intervention time
Bonhauser et al., 2005	PA-only	School-based physical activity program aimed at improving physical fitness.	90 min/session, 3 sessions/week; four 10-week units (120 sessions planned; 104 delivered, 87%).	None	—	100%
Bruneau-Chávez et al., 2025	PA-only	HIIT incorporated into Physical Education classes.	45 min/session, 2 sessions/week, 12 weeks (10-min warm-up, ~25-min HIIT, 10-min cool-down).	None	—	100%
Carrasco-Beltrán et al., 2015	PA-only	Moderate-to-vigorous small-sided games.	60 min of small-sided games within a 75-min session (10-min warm-up + 2 × 30-min game periods + 5-min inter-period rest), 2 sessions/week, 11 weeks.	None	—	100%
Díaz-Martínez et al., 2015	Multicomponent	Daily physical activity modules.	5 days/week for 5 months: 45-min sessions on 3 days/week and 30–40-min teacher-led physical activity on 2 days/week.	Healthy-eating/dietary education delivered to children and their families.	Duration of educational workshops not reported in comparable time units.	Not calculable from the published report
Galdames-Maliqueo et al., 2017	PA-only	HIIT based on maximal aerobic speed derived from the 20-m shuttle-run test.	16 sessions in total; session duration not clearly reported.	None	—	100%
Godoy-Cumillaf et al., 2024	Multicomponent (PA-promotion/ecological)	Moderate-to-vigorous games outside school hours plus in-school movement opportunities.	45 min/session, 3 sessions/week for 5 months; additional school-based movement opportunities were provided daily.	Educational activities to promote PA among parents, teachers, and children; weekly videos; school environmental movement strategies.	Parents: 15-min talk monthly; weekly short videos; teacher-led child activities 3 times/week; duration of several components not reported.	Not calculable from the published report
Kain et al., 2004	Multicomponent	Additional school PA, CALC, active recess, and sport-oriented activities.	Extra 90 min/week for 6 months (grades 3–8); CALC once/week; active recess 15 min/day for ~3 months; additional school-specific activities.	Nutrition education, parent involvement, school-kiosk/healthy-snack strategies, and other health-promotion activities.	Component durations varied and several strategies were not time-based or were incompletely reported.	Not calculable from the published report

Study	Intervention classification	Physical activity component	Physical activity dose	Other intervention component(s)	Dose of other component(s)	PA share of scheduled intervention time
Kain et al., 2008	Multicomponent	Additional PE/sports workshops, CALC, family PA activities, and teacher training to improve PE.	Extra 90 min/week over two school years (11 months); other PA components varied by year.	Nutrition education, parent talks, and community/healthy-eating activities.	Variable; some components reported as talks/activities rather than comparable minutes.	Not calculable from the published report
Kain et al., 2009	Multicomponent	Teacher-led PA promotion and training to improve the quality of PE classes; sports equipment provided.	5-month program; physical activity was integrated into the school program, but a single standardized child-level PA dose was not reported.	Healthy-eating education; in four schools, teachers also received individualized healthy-lifestyle counselling.	Teacher counselling: 3 sessions of ~15 min; child nutrition/education dose not fully reported.	Not calculable from the published report
Kain et al., 2012	Multicomponent	Increase in PE frequency and improvement in PE quality.	PE increased from 3 to 4 sessions/week, 45 min each; 3-year intervention.	Healthy-eating education, teacher training, and nutritionist supervision.	Child nutrition activities: 1 h every 15 days; teacher training: 9 h at the beginning of 2007 and 2008; monthly supervision.	Not calculable from the published report
Kain et al., 2014	Multicomponent	Increased PE class time and teacher training to improve PE quality and time in moderate-to-vigorous activity.	12-month intervention. In 2011, 2 weekly PE classes (90 + 45 min); in 2012, the second class increased to 90 min.	Classroom healthy-eating education, parent contact, teacher training, and school-kiosk/healthy-snack strategies.	Healthy-eating curriculum: 8 sessions of 90 min; parent contact: 3 × 15 min achieved; other components included teacher/kiosk training.	Not calculable from the published report
Lobos-Fernández et al., 2013	Multicomponent	Increase in PE time and use of specialist PE teachers.	PE increased from 3 to 4 h/week during a 2-year intervention.	Nutrition education, teacher training, and educational materials based on Chilean dietary guidelines.	Not fully reported in comparable time units.	Not calculable from the published report
Marín et al., 2011	Multicomponent	Structured physical activity/exercise sessions within a multidisciplinary childhood-obesity treatment program.	60 min/session, 2 sessions/week, 16 sessions in total (as extracted in the review dataset).	Nutritional and clinical/behavioral management components of the MINSAL-FONASA program.	Not fully reported in comparable time units in the available report.	Not calculable from the published report
Mellado-Peña et al., 2020	Multicomponent	Physical activity sessions within *Programa Vida Sana* 2017.	48–72 PA sessions recommended over 6 months.	Medical, nutritional, and psychological consultations; laboratory examinations; Healthy Life workshops.	Program recommended 2 psychological consultations and 3–5 Healthy Life workshops; durations were not reported in comparable time units.	Not calculable from the published report
Pino et al., 2018	PA-only	HIIT delivered within Physical Education classes.	15 min/session, 2 sessions/week, 12 weeks.	None	—	100%

Study	Intervention classification	Physical activity component	Physical activity dose	Other intervention component(s)	Dose of other component(s)	PA share of scheduled intervention time
Ratner et al., 2013	Multicomponent	Additional school physical activity delivered during recess.	Extra 90 min/week over a 2-year school-based program.	Nutrition education and healthy-kiosk intervention.	Nutrition education: 12 sessions of 45 min; kiosk component not time-comparable.	Not calculable from the published report
Soto-Sánchez et al., 2014	PA-only	Dynamic recreational games during the longest school recess.	45 min/session, 5 days/week, 12 weeks (3 months).	None	—	100%
Vásquez et al., 2013	Multicomponent	Supervised muscular-strength exercise at school.	45 min/session, 3 sessions/week, 30 sessions over 3 months.	Nutrition education and psychological support for children and parents.	6 nutrition sessions (5 child + 1 parent) and 6 psychology sessions (5 child + 1 parent); session duration not reported.	Not calculable from the published report

Notes: PA-only indicates that the planned intervention-specific exposure consisted exclusively of physical activity/exercise; usual Physical Education received by comparison groups was not counted as an intervention component. Multicomponent indicates that physical activity was combined with nutrition, psychological/health education, family/school strategies, or other program components. The PA-time proportion refers to the share of scheduled intervention time attributable to physical activity and was calculated only when all intervention components were reported in directly comparable time units. When one or more component durations were not reported, varied across participants or years, or involved non-time-based strategies (e.g., environmental or kiosk changes), the proportion was classified as "Not calculable from the published report" rather than estimated. Abbreviations: PA, physical activity; PE, Physical Education; HIIT, high-intensity interval training; CALC, Canadian Active Living Challenge.

Table S7. Sensitivity analyses by removing studies one by one from the main analyses.

Study excluded	SMD	LL	UL	I^2 (%)
**Body Mass Index** (uncontrolled before–after studies)				
Diaz Martínez et al. (2015)	-0.14	-0.39	0.12	99.0
Kain et al. (2009)	-0.15	-0.40	0.10	98.9
Kain et al. (2012)	-0.13	-0.38	0.13	98.9
Lobos Fernández et al. (2013)	-0.20	-0.40	-0.01	97.9
Mellado Peña et al. (2019)	-0.10	-0.35	0.14	98.9
Ratner et al. (2013)	-0.04	-0.19	0.11	98.3
Soto-Sanchez et al. (2014)	-0.14	-0.39	0.11	99.0
**Body Mass Index** (studies with a concurrent control group)				
Carrasco Beltrán et al. (2015)	-0.41	-0.89	0.07	93.0
Godoy-Cumillaf et al. (2024)	-0.45	-0.91	0.01	92.7
Kain et al. (2004)	-0.42	-0.90	0.07	92.4
Kain et al. (2008)	-0.40	-0.90	0.09	93.1
Kain et al. (2014)	-0.40	-0.90	0.09	93.0
Pino et al. (2018)	-0.17	-0.39	0.05	67.5
Vásquez et al. (2013)	-0.30	-0.78	0.17	91.9
**Waist Circumference** (studies with a concurrent control group)				
Godoy-Cumillaf et al. (2024)	-0.31	-0.90	0.27	92.3
Kain et al. (2004)	-0.33	-0.91	0.25	92.0
Kain et al. (2008)	-0.37	-0.90	0.16	89.4
Vásquez et al. (2013)	-0.04	-0.14	0.05	56.2
**Body fat percentage** (studies with a concurrent control group)				
Bruneau-Chávez et al. (2025)	-0.86	-1.61	-0.10	89.1
Carrasco Beltrán et al. (2015)	-1.03	-1.50	-0.56	81.3
Pino et al. (2018)	-0.62	-1.03	-0.20	63.2

Vásquez et al. (2013)	-0.80	-1.57	-0.04	89.8
**Cardiorespiratory fitness** (studies with a concurrent control group)				
Bruneau-Chávez et al., (2025)	0.68	0.41	0.96	67.3
Carrasco Beltrán et al., (2015)	0.79	0.48	1.10	77.2
Galdames-Maliqueo et al., (2017)	0.79	0.48	1.09	77.2
Godoy-Cumillaf et al. (2024)	0.75	0.42	1.08	77.4
Kain et al. (2004)	0.78	0.44	1.11	74.5
Kain et al. (2008)	0.85	0.57	1.12	71.3
Pino et al. (2018)	0.68	0.40	0.95	61.3

Notes: Pooled estimations after excluding from the analysis the corresponding reference. Leave-one-out sensitivity analyses were conducted for outcomes including at least three studies. Abbreviations: LL, lower limit; SMD, standard mean difference; UL, upper limit.

Table S8. Sensitivity analysis of BMI estimates using alternative pre–post correlations in uncontrolled before–after studies.

Assumed pre–post correlation	Pooled SMD	95% CI	I^2	$τ^2$	95% prediction interval
r = 0.30	−0.130	−0.348 to 0.088	98.3%	0.0837	−0.888 to 0.629
r = 0.50	−0.129	−0.346 to 0.088	98.8%	0.0837	−0.887 to 0.629
r = 0.70	−0.128	−0.344 to 0.088	99.2%	0.0836	−0.886 to 0.629

Abbreviations: BMI, body mass index; CI, confidence interval; SMD, standardized mean difference.
